# First insights on the genetic diversity of MDR *Mycobacterium tuberculosis* in Lebanon

**DOI:** 10.1186/s12879-018-3626-3

**Published:** 2018-12-29

**Authors:** Balig Panossian, Tamara Salloum, George F. Araj, Georges Khazen, Sima Tokajian

**Affiliations:** 1 Department of Natural Sciences, School of Arts and Sciences, Lebanese American University, Byblos Campus, P.O. Box 36, Byblos, Lebanon; 2 Department of Pathology and Laboratory Medicine, American University of Beirut Medical Center, Beirut, Lebanon; 3 Department of Computer Science and Mathematics, School of Arts and Sciences, Lebanese American University, Byblos, Lebanon

**Keywords:** *Mycobacterium tuberculosis*, WGS, SNPs, MDR-TB, End-TB

## Abstract

**Background:**

Lebanon hosts a heterogeneous population coming from underdeveloped and developing countries, resulting in increasing incidences of tuberculosis over the past years. The genetic heterogeneity and lineages associated with tuberculosis, along with their resistance determinants have not been studied at the genomic level previously in the region.

**Methods:**

Isolates were recovered from the American University of Beirut Medical Center (AUBMC). Antimicrobial susceptibility profiles were determined using the MGIT automated system for the first-line drugs at AUBMC, while second-line drug susceptibility was tested at Mayo Clinic Laboratories. Whole Genome Sequencing (WGS) was performed to classify mycobacterial lineages and highlight single nucleotide mutations causing resistance to both 1st line and 2nd line antimicrobials. wgSNP analysis provided insights on the phylogeny of the isolates along with spoligotyping and core genomic SNVs, IS*6110* insertion sites, and variable number tandem repeats (VNTR).

**Results:**

The analyzed isolates carry distinct resistance determinants to isoniazid, rifampicin, ethambutol, quinolones, and streptomycin. The isolates belonged to different lineages including the Euro/American lineage (Lineage 4) (53.8%), *M. bovis* (15.4%) and Delhi/Central Asia (Lineage 1) (15.4%), Beijing/East Asia (Lineage 2) (7.7%), and East Africa/Indian Ocean lineage (Lineage 3) (7.7%) showing great phylogenetic differences at the genomic level.

**Conclusions:**

The population diversity in Lebanon holds an equally diverse and uncharacterized population of drug resistant mycobacteria. To achieve the WHO “END-TB” milestones of 2025 and 2035, Lebanon must decrease TB incidences by 95% in the next decade. This can only be done through WGS-based patient centered diagnosis with higher throughput and genomic resolution to improve treatment outcomes and to monitor transmission patterns.

**Electronic supplementary material:**

The online version of this article (10.1186/s12879-018-3626-3) contains supplementary material, which is available to authorized users.

## Background

Tuberculosis (TB) has been a global health problem for millennia and still causes epidemics worldwide. According to the 2017 WHO Global TB Report, at least 10.4 million people fell ill with TB in 2016 [1]. For the past five years, it ranked as the leading cause of death from a single infectious agent, causing more mortalities than HIV/AIDS [1]. A major worldwide threat is the emergence of drug-resistant tuberculosis (DR-TB) and multidrug-resistant TB (MDR-TB) carrying resistance to both isoniazid and rifampicin and even extensively drug resistant TB (XDR-TB) [2]. TB comprises seven lineages [3]. Lineages 1 and 3 are limited to East Africa and parts of Asia. Lineage 2, known as the East-Asian lineage, includes the Beijing family of strains and predominates in East Asia. Lineage 4, known as the Euro-American lineage, is present in Asia, Europe, Africa and America while lineages 5–7 are geographically restricted to specific regions in Africa [4].

In Lebanon, incidences of TB are on the rise since 2006 with increasing mortality rates, especially among HIV-positive patients [1, 5]. A total of 860 people in Lebanon were infected with TB in 2016 [1]. With a lack of studies in Lebanon on TB epidemiology, burden and genetic diversity, there is a need to address TB influx patterns. Lebanon hosts, in addition to migrants from Syria, workers coming from Bangladesh, Pakistan, Philippines, Russian Federation, Vietnam, Ethiopia, India and other origins, which are among the highest 30 countries with MDR-TB incidences worldwide [1]. The genetic lineages of TB circulating in Lebanon were not addressed previously. Despite increasing incidences of TB in the country [1, 5], the “End TB Strategy” milestones set by the WHO are to reduce the mortality rates caused by TB by 35% in 2020, 75% in 2025 and by 95% in 2035 compared to the 2015 baseline values [1]. Thus, there is a definite need for more rapid and comprehensive diagnostics tools to improve treatment outcomes and epidemiological tracing to limit TB spread [6].

With decreasing costs, increasing availability and ease of usage [7], whole genome sequencing (WGS) provides unprecedented opportunities to obtain reliable predictions of drug susceptibilities and accurate identification of genetic polymorphisms such as single nucleotide polymorphisms (SNPs) and small insertions and deletions (indels) affecting drug resistance in TB [8, 9]. Analysis of genetic markers provided from WGS was previously used to distinguish persistent infections from MDR-TB and XDR-TB reinfections [10, 11]. Moreover, integrating WGS with patients’ information was successful in identifying transmission events and super-spreaders of infections [12–14]. It was also used to overcome the limited resolution obtained from the mycobacterial interspersed repetitive unit-variable number tandem repeat (MIRU-VNTR) molecular characterization method [15], and to highlight several outbreak scenarios that were previously missed by standard routine techniques [7]. End-to-end WGS-based diagnostic schemes and semi-automated pipelines for TB analysis have already shown promise in replacing traditional TB diagnostic tools in Europe and North America [16–18]. Another advantage of WGS is the direct sequencing of TB from sputum samples using culture-free approaches [19].

With the absence of genomic studies characterizing TB strains circulating in the Middle East, in this warranted pilot study, we performed WGS on 13 MDR-TB and susceptible-TB isolates collected from Lebanon to gain insights on the distribution of genetic lineages of TB, examine their genetic basis of drug resistance, and decipher mutations involved in antibiotic resistance genes. In order to gain a deeper understanding of the genomic diversity and evolution of TB in Lebanon, genomic profiling and phylogenetic analysis at the single nucleotide variants (SNV) level were conducted to accurately evaluate the position of local TB cases with respect to global reference genomes. The data obtained provides the first perceptions on the genetic composition of TB lineages circulating in the Middle East and confirms on the utility of WGS as a valuable technique in routine clinical diagnosis.

## Methods

### Sample collection

A total of 13 samples collected between 2015 and 2017 were provided by the Clinical Microbiology Laboratory (CML) of the Department of Pathology and Laboratory Medicine (PLM) at the American University of Beirut Medical Center (AUBMC), and were designated as TB4–5, TB7–10, TB-12-17 and TB20. The isolates were selected to include patients of various nationalities and represented a snapshot of the diversity of TB found in Lebanon. Specimens from patients suspected of having tuberculosis were cultured using Middlebrook 7H12 broth medium and Mycobacterial Growth Indicator Tubes (BACTEC 12B/ MGIT medium) in accordance with standard protocols for mycobacterial growth [20].

### Identification of mycobacterial isolates

The recovered isolates in conjunction with Acid Fast Smear were differentiated using BD MGIT TBc identification test (Becton Dickenson co. Wokingham Berkshire, UK.) to indicate MTB complex or MOTT (Mycobacteria other than tuberculosis).

### Phenotypic antimicrobial susceptibility testing

Phenotypic antimicrobial susceptibility profiles were determined against eight antibiotics: Isoniazid (0.1 μg/ml), rifampin(1.0 μg/ml), ethambutol (5.0 μg/ml), streptomycin (1.0 μg/ml),quinolones [ofloxacin (2.0 μg/ml), moxifloxacin (0.5 μg/ml, low concentration and 2.0 μg/ml, high concentration], amikacin (1.0 μg/ml), capreomycin (2.5 μg/ml), and kanamycin (2.5 μg/ml) using the automated Mycobacteria Growth Indicator Tube (MGIT) automated system for the first-line drugs available at AUBMC. Testing for the second-line drugs was carried out at Mayo Clinic Laboratories (Rochester, Minnesota, USA). A control strain of *M. tuberculosis* (H37Rv, ATCC 27294), was included in each run for quality control.

### DNA extraction

Mycobacterial cells were harvested from MGIT liquid media by centrifugation for 10 min at 7500 rpm. Each sample pellet was resuspended in a digestion buffer composed of 100 ul TE buffer, 3 ul Mutanolysin (5000 U/ml) (Sigma, Darmstadt, Germany), and 5 mg lysozyme (Sigma, Darmstadt, Germany) and incubated for 40 min at 37 °C on a shaking incubator. 100 ul PBS was added. 1 ul carrier RNA (Qiagen, Hilden, Germany) was added to 200 ul AL buffer (Qiagen, Hilden, Germany) and mixed into the sample. Subsequent steps were carried out using QIAamp DNA Blood Mini Kit (Qiagen, Hilden, Germany) according to the manufacturer’s guidelines.

### Whole genome sequencing

Library preparation was performed using the Illumina Nextera XT DNA library preparation kit (Illumina, San Diego, CA, USA). 1 μg of sample DNA was used as input for library preparation. The gDNA was subjected to end-repair, A-tailing, ligation of adaptors including sample-specific barcodes as recommended in the manufacturer’s protocol. The resulting library was quantified using Qubit 2.0 fluorometer (Invitrogen, Carlsbad, CA, USA). The library was sequenced on an Illumina MiSeq (Illumina, San Diego, CA, USA) with paired-end 500 cycles protocol to read a length of 250 bp.

### MTBC lineages/sub-lineages prediction

Total Genotyping Solution for *M. tuberculosis* (TGS-TB) online tool [21] (https://gph.niid.go.jp/tgs-tb/) was used for MTBC lineages/sub-lineages prediction using the KvarQ script according to the manufacturer’s instructions. The in silico detection of IS*6110* insertion, in silico spoligotyping and genotyping based on short MIRU-VNTRs was performed using TGS-TB [See Additional file 1] [22]. Sublineages were also generated and cross-validated using established tools TB Profiler [16] and Mykrobe Predictor-TB [18] [See Additional file 2].

### Core-genome phylogenetic and linkage network analysis

Adapters were trimmed and low quality bases with a Phred score less than 15 were discarded to obtain at least 50-mer length reads using Skewer [23] implemented in TGS-TB. The remaining reads were mapped against Mtb H37Rv [24] using BWA-mem program [25]. SNV sites having at least 5x coverage and a Phred score of at least 20 were used for mapping. Finding of additional SNVs sites among tested strains extracted by mpileup in SAMtools [26] after bwa mem mapping.

21,805 core genome SNVs on the non-repetitive regions [See Additional file 3], along with query-specific novel SNV sites were used to evaluate and identify the phylogenetic positioning of the isolates.

A maximum likelihood phylogenetic tree of all concatenated SNV alleles of the isolates and 78 reference genomes [21] was constructed on RAxML v8.2.0 [27] with 1000 bootstrap iterations.

The generated newick tree file was used in downstream visualization of the phylogenetic data using the Interactive Tree of Life tool [28] with external data sets for annotation of IS*6110*, spoligotypes, lineages, VNTRs, and antimicrobial resistance (AMR)-specific SNVs.

### In silico mapping of IS*6110* insertion sites

IS*6110* insertion sites were detected on TGS-TB [21] by collecting IS*6110* positions from short reads, rearranging the direction of the IS*6110* sequences, trimming and mapping the resulting reads to the Mtb H37Rv chromosome (accession # NC_000962.3) [24] using BWA-SW mapping [25].

### In silico Spoligotyping

In silico spoligotyping was performed through a Blastn search of 43 spacer sequences against the obtained short reads. Double mismatches with homology were considered a positive threshold [21, 29]. Spoligotypes were also generated and cross-validated using Spotyping [30] with identical results (data not shown).

### sMIRU-VNTR

VNTR loci were extracted from the H37Rv reference genome from the microorganisms tandem repeats database (http://minisatellites-rec.igmors.u-psud.fr/GPMS/). sMIRU-VNTR based on 43 discriminative loci were used to identify repeating units of short lengths flanked by tandem repeats. This approach made use of the short reads produced by Illumina sequencing instead of classical MIRU-VNTR MtbC15–9 type VNTR analysis as the latter requires fragments of up to 300 bp for accurate discrimination between samples.

The chosen loci, their nucleotide positions on the H37Rv reference genome, and additional information as previously described are summarized in [See Additional file 4}.

MIRU-VNTR patterns of the 24 classical loci were also generated using MIRUprofiler [31].

### Antimicrobial resistance genes

Raw reads were used to directly identify key antimicrobial resistance profiles of the isolates based on characteristic mutations and their frequencies in the reads using Mykrobe Predictor-TB [18] (https://github.com/iqbal-lab/Mykrobe-predictor/releases)) Version:MTBC.niid.3.20150403.

Mutations conferring AMR were identified and annotated based on the improved target list of KvarQ [22]. Alternative alleles of drug resistance SNPs were identified using TB profiler [16] [See Additional file 5]. Resistance encoding mutations were cross-validated using 3 softwares: KvarQ, Mykrobe predictor, and TB profiler [16] with identical results obtained.

## Results

### Diversity of MTBC lineages

In total, 13 cases were included in this study; 46% (*n* = 6) were males and 54% (*n* = 7) were females. The patients’ age ranged from 29 to 57 with an average of 43.3 years. 69.2% (*n* = 9) of the isolates were obtained from sputum, 15.4% (*n* = 2) from tissue cultures, 7.7% (*n* = 1) from an abscess, and 7.7% (*n* = 1) from pleural fluid. The majority of the isolates were collected from Lebanese patients, making 53.8% (n = 7) of the total followed by non-Lebanese patients from Iraq (15.4%; *n* = 2), Syria (7.7%; *n* = 1), the Philippines (7.7%; *n* = 1), Ethiopia (7.7%; *n* = 1) and Ukraine (7.7%; *n* = 1).

**Fig. 1:**
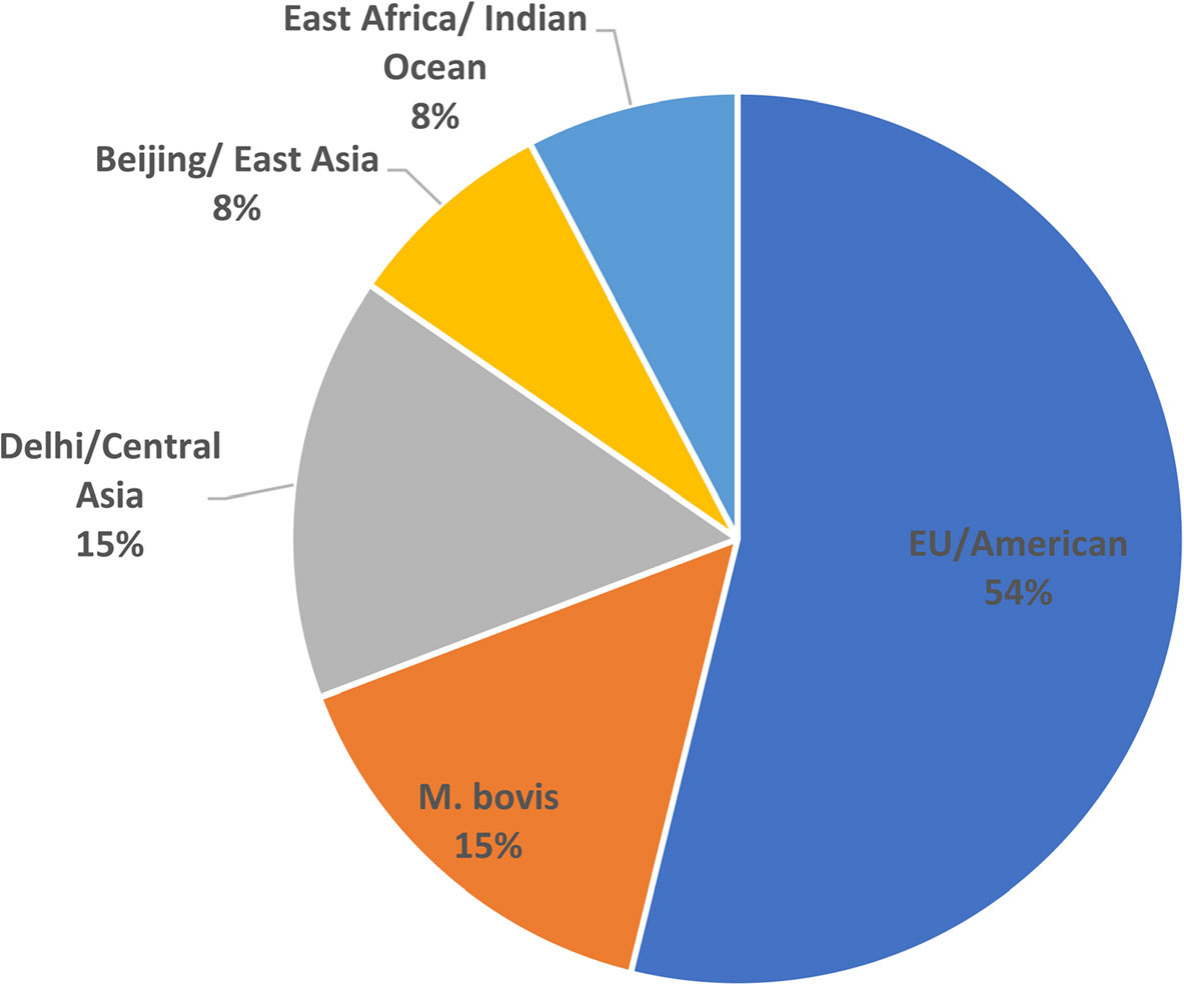
Distribution of TB lineages. Detailed Legend: Pie chart showing the distribution of mycobacterial lineages in the collected samples

The isolates were not from a single source, but instead belonged to different lineages. The most common lineage was the Euro/American lineage (Lineage 4) (53.8%; *n* = 7), followed by *M. bovis* (15.4%; *n* = 2), the Delhi/Central Asia lineage (Lineage 1) (15.4%; *n* = 2), the Beijing/East Asia lineage (Lineage 2) (7.7%; *n* = 1), and the East Africa/Indian ocean lineage (7.7%; *n* = 1) (Lineage 3) (Fig. 1).

### Phylogenetic relatedness and antimicrobial resistance

**Fig. 2:**
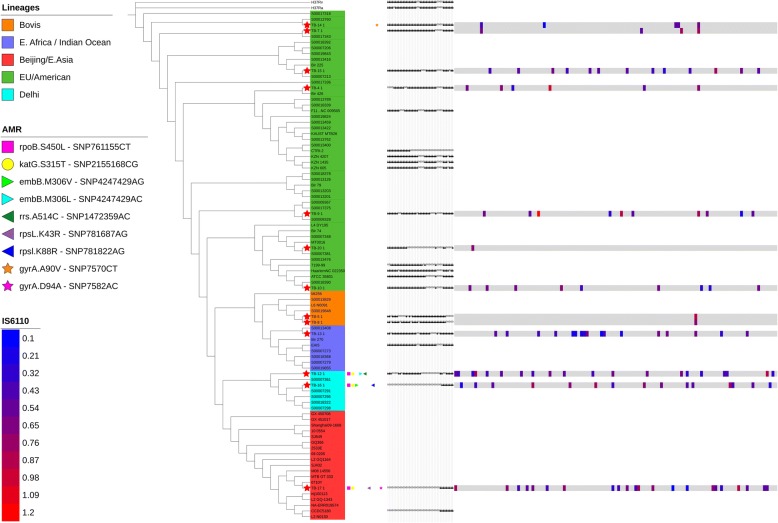
Linear phylogenetic tree showing wgSNP, AMR conferring SNPs and IS6110 sites. Detailed Legend: Maximum-likelihood linear phylogenetic tree based on core genome phylogeny of the samples. Branches show sample distribution in all international lineages, along with spoligotypes and IS*6110* insertion sites and AMR conferring SNPs

A maximum likelihood phylogenetic tree of all concatenated SNV alleles of the isolates was constructed along with 78 reference genomes covering all lineages including H37Rv and H37Ra. The isolates were grouped into specific clades in agreement with their assigned lineages, and were highlighted accordingly (Fig. 2).

The European/American lineage (Lineage 4) consisted of seven Mtb isolates (TB-4, TB-7, TB9, TB-10, TB-14, TB-15 and TB-20). These isolates were susceptible to all the tested antibiotics except for TB-14 that was resistant to ofloxacin and moxifloxacin quinolones. Out of the European/American lineage, 71.4% (*n* = 5) were collected from patients of Lebanese origin, 14.3% (*n* = 1) from Syria and 14.3% (*n* = 1) from Iraq. A threshold of 5 SNPs between TB isolates has been proposed as an indicator of recent transmission between patients while > 12 SNPs is considered to be evidence against transmission [32, 33].

Three of the studied isolates (23%) were MDR (TB-12, TB-16 and TB-17). Detected mutations in genes conferring resistance to isoniazid, rifampicin, ethambutol, quinolones and streptomycin accurately predicted the obtained drug resistant phenotypes. The isolates’ resistance patterns and SNVs corresponding to each AMR gene are represented in Additional file 5.

One of the MDR isolates, TB-17, clustered under the Beijing/East Asia lineage (Lineage 2) and was collected from a Ukrainian patient while the two other MDRs, TB-12 and TB-16, clustered under the Delhi/Central Asia lineage (Lineage 1) and were collected from Iraqi and Ethiopian patients, respectively. The East African/Indian Ocean lineage (Lineage 3) included TB-13 collected from a Filipino patient. TB-5 and TB-8 belonged to the *M. bovis* lineage and were both obtained from Lebanese patients. A closer inspection of the two pairs of seemingly related isolates, namely TB12 and TB16 as well as TB5 and TB8, revealed great variability in their core genome SNVs. 361 core genome SNVs were identified between TB12 and TB16, while TB5 and TB8 differed by 625 core genome SNVs.

### IS*6110* insertion sites

In silico analysis of IS*6110* insertion sites was performed. The positions of IS*6110* was in agreement with different assigned lineages except for two isolates belonging to lineage 4: TB-15 was closely associated with TB-17 (Lineage 2), and TB-10 was closely associated with TB-13 (Lineage 3) and TB-5 and TB-8 (*M. bovis* lineage).

Lineage specific preferential insertion sequences regions of IS*6110* were examined as previously described by Roychowdhury et al. [34]. As for the MDR isolate TB-17, which belonged to Beijing/East Asia lineage (Lineage 2), 20 IS*6110* in total were identified. Out of these, six belonged to Lineage 2 specific insertion regions (Rv1371, Rv1765-Rv1765A, Rv3018-Rv3019, Rv3179-Rv3180, Rv3383c (*idsB*) and Rv3427c transposase). The two MDR isolates belonging to the Delhi/Central Asia lineage (Lineage 1), TB-12 and TB-16 had different IS*6110* profiles, whereas 18 positions were detected in TB-12 and 14 positions in TB-16 (Fig. 2).

### sMIRU-VNTR

**Fig. 3:**
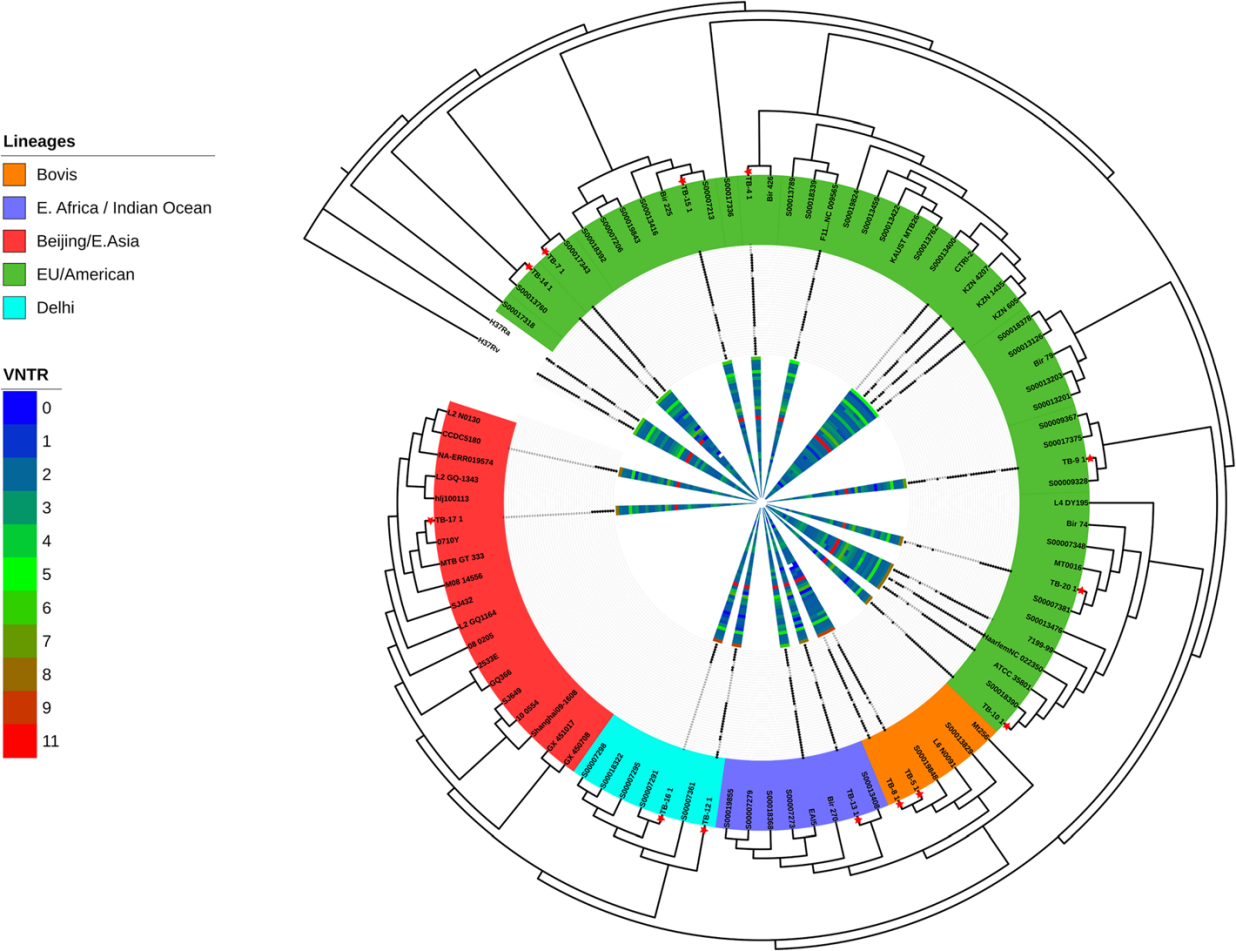
Circular Tree of TB phylogeny with wgSNP, VNTR and Spoligotype data. Detailed Legend: Maximum-likelihood phylogenetic tree based on core genome phylogeny of the samples with 1000 bootstrap iterations. Circular tree with inverted branches shows sample distribution in all international lineages, along with spoligotypes and VNTR data. Isolates from our study are annotated with red stars

sMIRU-VNTR retained a general pattern across all samples with characteristic hotspots, annotated Green and Red on the VNTR heatmap (Fig. 3) at positions 5, 14, 16, 19, 30, 31, and 36 of the 43 sMIRU-VNTR loci analyzed.

The results of MIRUprofiler of the 24 classical VNTR loci are summarized in the Additional file 6.

### Spoligotyping

In silico clustering based on spoligotypes was in accordance with IS*6110* mapping and assigned lineages. The isolates had different patterns except for TB-7 and TB-14 that differed by a single spacer oligonucleotide at position 32. TB-17 shared an identical pattern to *M. tuberculosis* CCDC5180 reference strain (accession # NC_017522) both belonging to the Beijing lineage. Similarly, TB-10 and *M. tuberculosis* Erdman (ATCC35801) reference strain (accession # NC_020559) shared identical patterns and both belonged to the EU/American lineage [See Additional file 1].

The isolates undertaken in this study were annotated with a red star, and were situated in between international reference strains of notable significance.

## Discussion

This is the first report on the diversity of *M. tuberculosis* lineages circulating in Lebanon and in the region. In our collection of isolates, we identified types covering the most common global lineages, which revealed the heterogeneity within TB causing isolates in Lebanon. Our findings reveal that the Euro/American lineage (Lineage 4) was the most dominant in Lebanon (53.8%), followed by the *M. bovis* (15.4%) and Delhi/Central Asia (Lineage 1) (15.4%), the Beijing/East Asia (Lineage 2) (7.7%), and the East Africa/Indian Ocean lineage (Lineage 3) (7.7%). SNV-based phylogenetic analysis grouped the isolates in agreement with their assigned lineages with a higher accuracy than IS*6110* mapping and spoligotyping. The European/American lineage was found to be prevalent in several North African countries including Morocco, Tunisia, Algeria and Libya [35].

The MDR isolates belonged to the Beijing/East Asia lineage (Lineage 2) (TB-17), while the two other MDRs belonged to the Delhi/Central Asia lineage (Lineage 1) (TB-12 and TB-16). In fact, TB belonging to the Beijing/East Asian lineage was found to be driving the massive spread of MDR TB in Eurasia [36, 37]. They were associated with large MDR TB outbreaks [38] and appear to be rapidly expanding in population size [39] due to their hypermutability [40], increased transmissibility, and hypervirulence compared to other MTBC lineage [41]. On the other hand, the Delhi/Central Asia lineage is the second predominant genotype in pre-XDR and XDR TB following the Beijing/East Asian lineage [42]. The Delhi/Central Asia lineage was detected in the Middle East and Central Asia and preferentially in India [43].

In this study, 15.4% (*n* = 2) of the isolates belonged to the *M. bovis* lineage (TB-5 and TB-8) and had different spoligotypes. They showed one IS*6110* compared to an average of 9.5 IS*6110* in the other isolates. IS*6110* copy number ranges from 0 to 27, with a mean of approximately 13 in the seven TB lineages [34]. Differing levels of IS could be related to TB host adaptability [44]. Yet, to date, IS6*110* copy number was not explored in *M. bovis* although it was considerably less than that in *M. tuberculosis*, with typically just one insertion noted [45], as also observed in our study. Interestingly, a unique single transposable IS*6110* upstream of the *phoPR* operon increased its expression, thereby facilitating the dissemination of *M. bovis* in the human host [46]. Scarce data on *M. bovis* exist in Lebanon with the prevalence being 3.3% [47]. The consumption of raw milk appears to be a common source of transmission [48], and the burden of *M. bovis* is underestimated in human beings [49]. Symptomatic and asymptomatic animals serve as reservoirs of *M. bovis* and favor its dissemination to humans and wildlife species [50]. The accurate diagnosis and treatment of *M. bovis* in humans is necessary to achieve a world free of TB by 2035 as part of the WHO’s end TB strategy [51].

Multidrug-Resistant TB (MDR-TB) isolates belonging to the Delhi (*n* = 2) lineage and the Beijing (*n* = 1) lineage clustered significantly far from one another based on their phylogenetic SNV grouping. Two SNPs, noted as SNP761155CT and SNP2155168CG were detected in all three of the MDR isolates. These characteristic point mutations are causative of changes in *rpoB* S450 L and *katG* S315 T respectively. De novo generation of drug resistant TB is a major healthcare concern, as the incidence, treatment days, treatment expenses, and mortality rates would all increase with the spontaneous generation of resistant strains immune to the very few available effective antimicrobial agents.

By comparing the spoligotype and VNTR fingerprints of the isolates with the SNV phylogenetic clustering, some of the isolates in this study showed nearly identical VNTR patterns and spoligotype fingerprints yet appeared to be phylogenetically unrelated. Also, the SNV-based phylogenetic analysis was also more accurate in grouping isolates to lineages compared to IS*6110* that in the case of our isolates did not accurately assign two isolates belonging to lineage 4. This revealed the presence of genome-based differences at regions not linked to those targeted by the aforementioned classical typing methods. Other advantages of WGS include outbreak analysis and surveillance [52] and the sequencing from uncultured sputa of patients with diagnosis-ready data generated in just a few days from sample collection [19].

Although the small number of studied samples provided some insights into the heterogeneity of TB circulating in Lebanon, it may not represent all the genetic diversity of TB in the region and the collection of a larger sample size is necessary. Thus, our efforts will include a broader sampling that includes Lebanon and neighboring countries. As a further step forward, we push for the analysis of the intra-host evolution of MDR-TB isolates to prevent treatment failure and decrease mortality rates. Reinfection by MDR-TB or relapse is of critical importance, thus the detection of de novo generated SNVs especially in HIV positive patients is of paramount importance [53].

## Conclusion

As highlighted very recently, the burden of TB has been a major public health issue in Lebanon. The Syrian humanitarian crisis completes its 8th year in succession, continuously adding to the weight of the problem at hand [54]. With over 600 new cases arising per year in the last decade, and the scarcity of studies that shed light on the evolution of TB in the region [5], Lebanon has been held back from joining the global efforts to fight TB [1]. Taking into account the small size of Lebanon, the genomic heterogeneity of mycobacteria can only be understood and better dealt with by high quality differentiation and high throughput methods.

If we are to decrease incidences to the WHO specified pre-elimination limit of < 10 TB cases per million by 2035, and < 1 TB case per million by 2050, TB incidences have to drop by 90% in the following decade, and by 99% in the year 2050 [1]. Thus, we recommend more stringent TB testing of foreign workers prior to their travel to Lebanon, and push towards the implementation of WGS as a core diagnostic tool for TB management over the coming years.

## Additional files

Additional file 1: Results of TGS-TB for in silico detection of IS6110 insertion sites, spoligotyping based on 43 spacer oligos, and genotyping based on 43 sMIRU-VNTRs. The red and sky-blue vertical bars indicate the forward and reverse IS*6110* insertions; filled circles indicate positive homology to each oligo; the detected number of TRs is shown on each locus and visualized using a color variation scale, black and grey boxes indicate no detection of TRs and lower depths, respectively. (PDF 860 kb)

Additional file 2: List of *M. tuberculosis* strains used in this study. Lineages/Sublineages generated using TGS-TB and TB Profiler. Table containing a list of the TB strains used with Genbank ID and Lineage of each used for the phylogenetic analysis of the isolates on the TGS-TB web server and sublineages as predicted by TGS-TB and TB Profiler. (PDF 63 kb)

Additional file 3: Repeat regions on *M. tuberculosis* H37Rv genome. Repeat regions of H37Rv excluded from the analysis of 21,805 core genome SNVs which was done on the non-repetitive regions done on the TGS-TB web server. (PDF 65 kb)

Additional file 4: Loci Information for short MIRU-VNTR. List of Loci taken into account for the analysis of the short mycobacterial interspersed repeat units variable number of tandem repeats (sMIRU-VNTR) analysis done on the TGS-TB web server. (PDF 197 kb)

Additional file 5: Antimicrobial resistance profiles and detected mutations in the corresponding AMR genes of the TB isolates. R: Resistant; S: sensitive; +: MDR; −: Not MDR; Sensitive and resistant are indicated in green and red, respectively. (PDF 37 kb)

Additional file 6: Results of the MIRU-Profiler showing the number of tandem repeats for the 24 classical MIRU loci. The results of MIRUprofiler are based on 24 classical VNTR loci. (PDF 13 kb)

## Abbreviations

AMR: Antimicrobial Resistance
AUBMC: American University of Beirut Medical Center
CML: Clinical Microbiology Laboratory
HIV: Human Immunodeficiency Virus
MDR-TB: Multidrug Resistant Tuberculosis
MGIT: Mycobacterial Growth Indicator Tube
PLM: Pathology and Laboratory Medicine
SNV: Single Nucleotide Variant
TB: Tuberculosis
TGS-TB: Total Genotyping Solution for *Mycobacterium tuberculosis*
VNTR: Variable Number of Tandem Repeats
WGS: Whole Genome Sequencing
wgSNP: Whole genome based Single Nucleotide Polymorphism analysis
WHO: World Health Organisation
XDR-TB: Extensively Drug Resistant Tuberculosis
