# Supplementary material for: First insights on the genetic diversity of MDR Mycobacterium tuberculosis in Lebanon
Source: BMC Infect Dis. 2018 Dec 29;18:710. doi: 10.1186/s12879-018-3626-3 (PMC6311033; doi:10.1186/s12879-018-3626-3)

IS6110 insert positions

Depth filter:

30 % Default

Lightblue: forward, Red: reverse
Length shows % depth for average.
Dots show number of the insertions
(Neighboring dots are shown at different vertical positions)

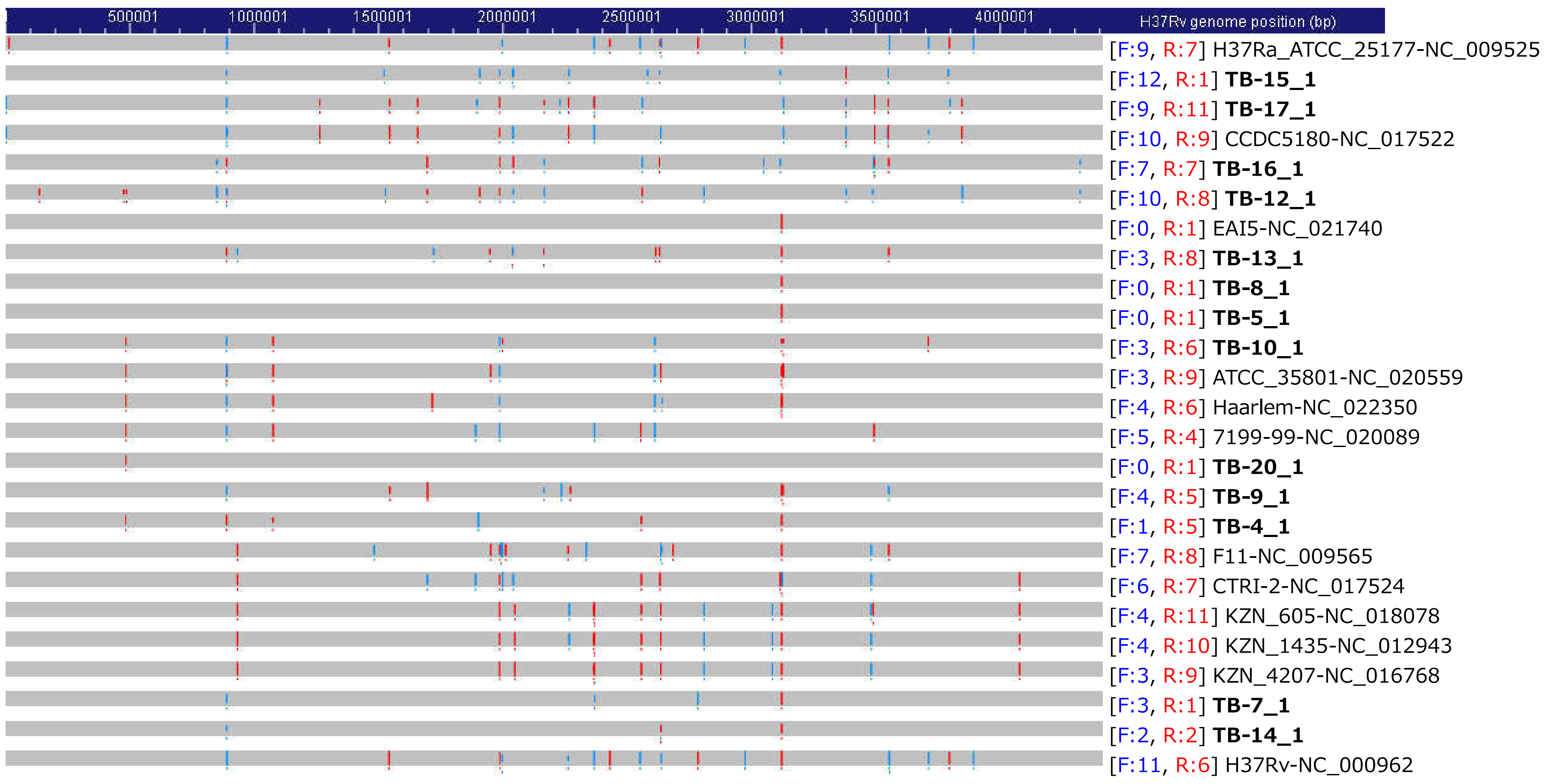

Spoligotyping

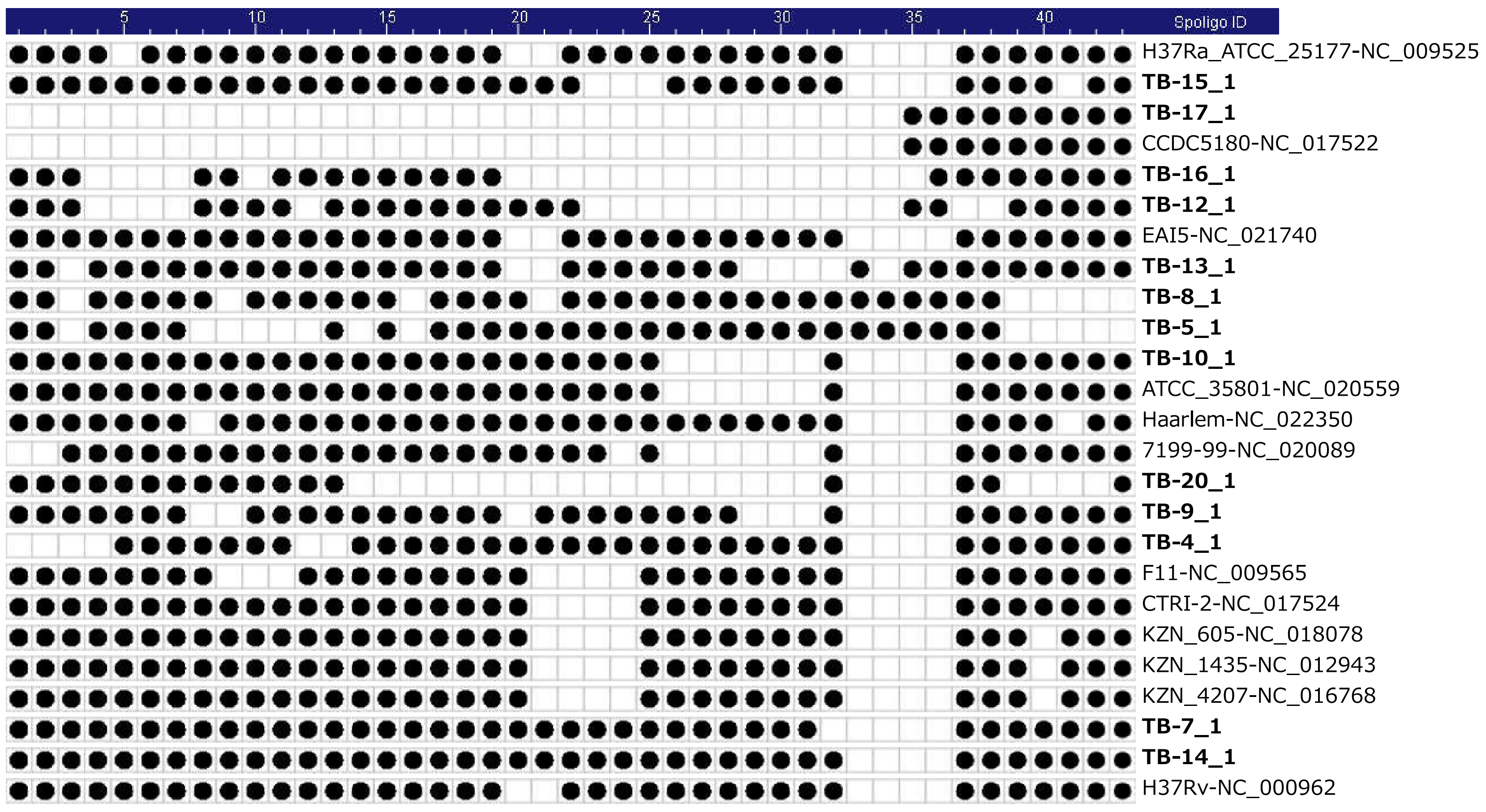

VNTR

Download our customised 43 VNTR loci list in PDF or Excel format.

Coloring setting of TRs ◉ Rainbow color ○ Monotone

Min: 1 Max: 11

Depth Filter: 10 %

Default

Gray box (▩) indicates lower than the depth filter setting (%).

Black box (■) indicates no detection TR.

Stripe patterned box (▨) indicates possible mixed number of TR.

Example

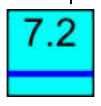

Number: Tandem repeats (TRs).
Blue horizontal line: Read depth ratio to avg. depth.
Box color: Corresponds to the TRs.
Lower than the depth filter setting.

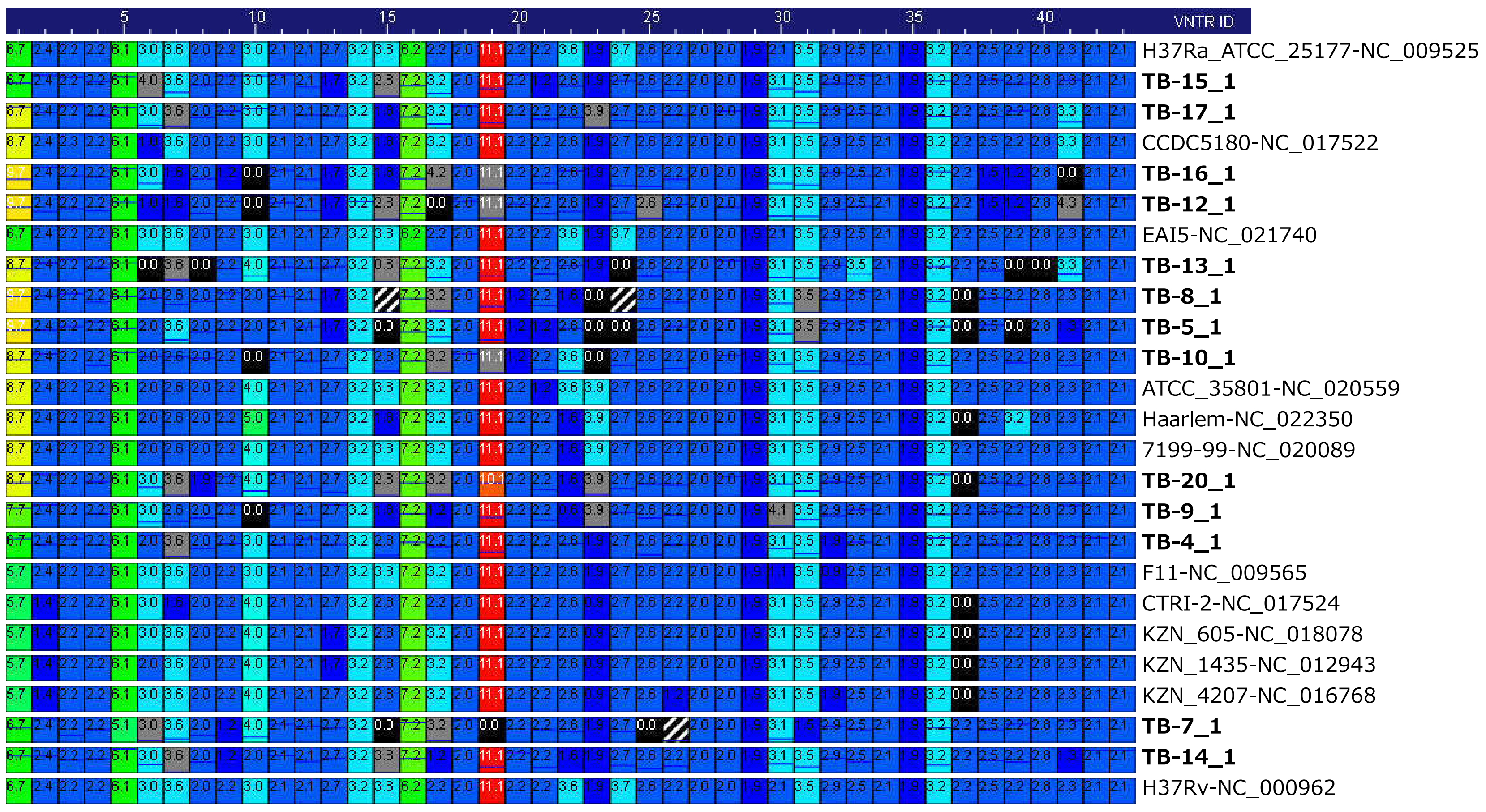

Supplement: Supplementary file 1 — Results of TGS-TB for in silico detection of IS6110 insertion sites, spoligotyping based on 43 spacer oligos, and genotyping based on 43 sMIRU-VNTRs. The red and sky-blue vertical bars indicate the forward and reverse IS6110 insertions; filled circles indicate positive homology to each oligo; the detected number of TRs is shown on each locus and visualized using a color variation scale, black and grey boxes indicate no detection of TRs and lower depths, respectively. (PDF 860 kb) [file 12879_2018_3626_MOESM1_ESM.pdf]
