# Supplementary material for: First insights on the genetic diversity of MDR Mycobacterium tuberculosis in Lebanon
Source: BMC Infect Dis. 2018 Dec 29;18:710. doi: 10.1186/s12879-018-3626-3 (PMC6311033; doi:10.1186/s12879-018-3626-3)
Supplement: Supplementary file 2 — List of M. tuberculosis strains used in this study. Lineages/Sublineages generated using TGS-TB and TB Profiler. Table containing a list of the TB strains used with Genbank ID and Lineage of each used for the phylogenetic analysis of the isolates on the TGS-TB web server and sublineages as predicted by TGS-TB and TB Profiler. (PDF 63 kb) [file 12879_2018_3626_MOESM2_ESM.pdf]

List of *M. tuberculosis* strains used in this study		
**Lineages/Sublineages generated using TGS-TB and TB Profiler**		
Strains ID	SRA_ID or GenBank_ID	Lineage
TB-4	PVWR00000000	4
TB-5	PYPT00000000	BOV
TB-7	PYPU00000000	4.7
TB-8	PYPV00000000	BOV
TB-9	PYPW00000000	4.2.1
TB-10	PYPX00000000	4.1.2.1
TB-12	PYPY00000000	3
TB-13	PYPZ00000000	1.2.1
TB-14	PYQA00000000	4.8
TB-15	PYQB00000000	4.6.2.2
TB-16	PYQC00000000	3.1.1
TB-17	PYQD00000000	2.2.1
TB-20	PYQE00000000	4.1.1.1
S00007279	ERR117694	1.1.1
S00019855	ERR387008	1.1.1.1
EAI5	NC_021740	1.1.2
S00007273	ERR114427	1.1.2
S00018368	ERR351915+ERR386847	1.1.3
S00013408	ERR278529+ERR386888	1.2.1
Bir_270	ERR046881	1.2.2
GX_450708	SRR1710066	2.1
GX_451017	SRR1710070	2.1 proto-Beijing
hlj100113	SRS475367	2.2.1
CCDC5180	NC_017522	2.2.1
08_0205	SRR1710073	2.2.1 MG2
SJ432	SRR1710110	2.2.1 MG3
0710Y	ERR117454	2.2.1 MG3
M08_14556	ERR015616	2.2.1 MG3
MTB_GT_333	ERR234208	2.2.1 MG3
-NA-	ERR019574	2.2.1 MG3
2533E	ERR234658	2.2.1 PG1
GQ366	ERR234133	2.2.1 PG1
L2_GQ1164	ERR234116	2.2.1 PG2
L2_GQ-1343	ERR234121	2.2.1.1
L2_N0130	ERR234263	2.2.1.2
10_0554	SRR1710083	2.2.2
Shanghai_09-1608	SRS790114	2.2.2 MG1
SJ649	SRR1710111	2.2.2 MG1
S00007361	ERR114513	3
S00007291	ERR114453	3.1.1

S00018322	ERR351869+ERR386801	3.1.2
S00007295	ERR117696	3.1.2.1
S00007298	ERR114442	3.1.2.2
H37Rv	NC_000962	4
S00017318	ERR330651	4
H37Ra_ATCC_25177	NC_009525	4
L4_DY195	ERR234201	4.1
Bir_74	ERR038277	4.1.1
S00007381	ERR114477	4.1.1.1
MT0016	SRR058116	4.1.1.2
S00007348	ERR117732	4.1.1.3
S00013476	ERR278597+ERR386956	4.1.2
7199-99	NC_020089	4.1.2.1
Haarlem	NC_022350	4.1.2.1
ATCC_35801	NC_020559	4.1.2.1
S00018390	ERR351937+ERR386869	4.1.2.1
S00009328	ERR228183	4.2.1
S00017375	ERR330708	4.2.2
S00009367	ERR228222	4.2.2.1
S00019824	ERR386977	4.3.1
S00013789	ERR294209	4.3.2
S00018339	ERR351886+ERR386818	4.3.2.1
F11	NC_009565	4.3.2.1
S00013400	ERR278521+ERR386880	4.3.3
CTRI-2	NC_017524	4.3.3
KZN_4207	NC_016768	4.3.3
KZN_605	NC_018078	4.3.3
KZN_1435	NC_012943	4.3.3
S00013459	ERR278580+ERR386939	4.3.4
S00013422	ERR278543+ERR386902	4.3.4.1
FFUL_KAUST_MTB26	ERR275206	4.3.4.2
S00013762	ERR294182	4.3.4.2.1
S00018378	ERR351925+ERR386857	4.4
S00013201	ERR270699	4.4.1
S00013203	ERR270701	4.4.1.1
Bir_79	ERR038282	4.4.1.2
S00013126	ERR270624	4.4.2
S00017336	ERR330669	4.5
S00018392	ERR351939+ERR386871	4.6
S00019843	ERR386996	4.6.1.1
S00007206	ERR123926	4.6.1.2
S00013416	ERR278537+ERR386896	4.6.2
Bir_225	ERR046839	4.6.2.1

S00007213	ERR123915	4.6.2.2
S00017343	ERR330676	4.7
S00013760	ERR294180	4.8
Bir_426	ERR072039	4.9
S00013829	ERR294249	5
L6_N0091	ERR234254	6
S00019848	ERR387001	BOV
Mt256	ERR181435	7
