# Supplementary material for: First insights on the genetic diversity of MDR Mycobacterium tuberculosis in Lebanon
Source: BMC Infect Dis. 2018 Dec 29;18:710. doi: 10.1186/s12879-018-3626-3 (PMC6311033; doi:10.1186/s12879-018-3626-3)
Supplement: Supplementary file 3 — Repeat regions on M. tuberculosis H37Rv genome. Repeat regions of H37Rv excluded from the analysis of 21,805 core genome SNVs which was done on the non-repetitive regions done on the TGS-TB web server. (PDF 65 kb) [file 12879_2018_3626_MOESM3_ESM.pdf]

S3 Table. Repeat regions on M. tuberculosis H37Rv genome

Start		End	Note
23173	-	23273	H37Rv annotation (NC_000962.3)
79507	-	79551	H37Rv annotation (NC_000962.3)
80236	-	80550	H37Rv annotation (NC_000962.3)
103713	-	105215	H37Rv annotation (NC_000962.3)
154073	-	154125	H37Rv annotation (NC_000962.3)
154126	-	154178	H37Rv annotation (NC_000962.3)
154179	-	154231	H37Rv annotation (NC_000962.3)
206812	-	206850	H37Rv annotation (NC_000962.3)
206869	-	206907	H37Rv annotation (NC_000962.3)
272855	-	272955	H37Rv annotation (NC_000962.3)
547488	-	547517	H37Rv annotation (NC_000962.3)
580578	-	580654	H37Rv annotation (NC_000962.3)
580655	-	580731	H37Rv annotation (NC_000962.3)
580732	-	580808	H37Rv annotation (NC_000962.3)
616828	-	616878	H37Rv annotation (NC_000962.3)
642754	-	642811	H37Rv annotation (NC_000962.3)
701247	-	701369	H37Rv annotation (NC_000962.3)
703912	-	703985	H37Rv annotation (NC_000962.3)
706790	-	706863	H37Rv annotation (NC_000962.3)
709425	-	709548	H37Rv annotation (NC_000962.3)
709585	-	709663	H37Rv annotation (NC_000962.3)
711624	-	711702	H37Rv annotation (NC_000962.3)
795467	-	795518	H37Rv annotation (NC_000962.3)
802429	-	802477	H37Rv annotation (NC_000962.3)
812835	-	812921	H37Rv annotation (NC_000962.3)
812922	-	812975	H37Rv annotation (NC_000962.3)
863155	-	863255	H37Rv annotation (NC_000962.3)
889017	-	889020	H37Rv annotation (NC_000962.3)
889021	-	889048	H37Rv annotation (NC_000962.3)
890348	-	890375	H37Rv annotation (NC_000962.3)
890376	-	890379	H37Rv annotation (NC_000962.3)
960173	-	960225	H37Rv annotation (NC_000962.3)
960226	-	960278	H37Rv annotation (NC_000962.3)
960279	-	960333	H37Rv annotation (NC_000962.3)
1025458	-	1025472	H37Rv annotation (NC_000962.3)
1026879	-	1026893	H37Rv annotation (NC_000962.3)
1027061	-	1027076	H37Rv annotation (NC_000962.3)
1029345	-	1029360	H37Rv annotation (NC_000962.3)
1164572	-	1164589	H37Rv annotation (NC_000962.3)
1165532	-	1165549	H37Rv annotation (NC_000962.3)
1179345	-	1179395	H37Rv annotation (NC_000962.3)
1251621	-	1252945	H37Rv annotation (NC_000962.3)
1276296	-	1277643	H37Rv annotation (NC_000962.3)
1277843	-	1277846	H37Rv annotation (NC_000962.3)
1277847	-	1277863	H37Rv annotation (NC_000962.3)
1278800	-	1278816	H37Rv annotation (NC_000962.3)
1278817	-	1278820	H37Rv annotation (NC_000962.3)
1305495	-	1305556	H37Rv annotation (NC_000962.3)
1305557	-	1305618	H37Rv annotation (NC_000962.3)
1305619	-	1305661	H37Rv annotation (NC_000962.3)
1456585	-	1456627	H37Rv annotation (NC_000962.3)
1457453	-	1457504	H37Rv annotation (NC_000962.3)
1457505	-	1457557	H37Rv annotation (NC_000962.3)
1468143	-	1468161	H37Rv annotation (NC_000962.3)
1469633	-	1469651	H37Rv annotation (NC_000962.3)
1507531	-	1507581	H37Rv annotation (NC_000962.3)
1541949	-	1541951	H37Rv annotation (NC_000962.3)

1543307	-	1543309	H37Rv annotation (NC_000962.3)
1612558	-	1612578	H37Rv annotation (NC_000962.3)
1612579	-	1612599	H37Rv annotation (NC_000962.3)
1612600	-	1612620	H37Rv annotation (NC_000962.3)
1612621	-	1612641	H37Rv annotation (NC_000962.3)
1612642	-	1612662	H37Rv annotation (NC_000962.3)
1625366	-	1625418	H37Rv annotation (NC_000962.3)
1633531	-	1634790	H37Rv annotation (NC_000962.3)
1637133	-	1638392	H37Rv annotation (NC_000962.3)
1644261	-	1644313	H37Rv annotation (NC_000962.3)
1644314	-	1644364	H37Rv annotation (NC_000962.3)
1779266	-	1779277	H37Rv annotation (NC_000962.3)
1779959	-	1780047	H37Rv annotation (NC_000962.3)
1780485	-	1780573	H37Rv annotation (NC_000962.3)
1788514	-	1789811	H37Rv annotation (NC_000962.3)
1788514	-	1788525	H37Rv annotation (NC_000962.3)
1830074	-	1830125	H37Rv annotation (NC_000962.3)
1907460	-	1907515	H37Rv annotation (NC_000962.3)
1907516	-	1907571	H37Rv annotation (NC_000962.3)
1927218	-	1928589	H37Rv annotation (NC_000962.3)
1938093	-	1938145	H37Rv annotation (NC_000962.3)
1944756	-	1944808	H37Rv annotation (NC_000962.3)
1982887	-	1982964	H37Rv annotation (NC_000962.3)
1982965	-	1983042	H37Rv annotation (NC_000962.3)
1983043	-	1983120	H37Rv annotation (NC_000962.3)
1983121	-	1983198	H37Rv annotation (NC_000962.3)
1983199	-	1983276	H37Rv annotation (NC_000962.3)
1983277	-	1983354	H37Rv annotation (NC_000962.3)
1987703	-	1987730	H37Rv annotation (NC_000962.3)
1989030	-	1989057	H37Rv annotation (NC_000962.3)
1996101	-	1996128	H37Rv annotation (NC_000962.3)
1997428	-	1997455	H37Rv annotation (NC_000962.3)
1998584	-	1998597	H37Rv annotation (NC_000962.3)
1999800	-	1999813	H37Rv annotation (NC_000962.3)
2059441	-	2059498	H37Rv annotation (NC_000962.3)
2059518	-	2059575	H37Rv annotation (NC_000962.3)
2163323	-	2163392	H37Rv annotation (NC_000962.3)
2163393	-	2163461	H37Rv annotation (NC_000962.3)
2163462	-	2163530	H37Rv annotation (NC_000962.3)
2163741	-	2163809	H37Rv annotation (NC_000962.3)
2163810	-	2163878	H37Rv annotation (NC_000962.3)
2163879	-	2163947	H37Rv annotation (NC_000962.3)
2163948	-	2164016	H37Rv annotation (NC_000962.3)
2164017	-	2164085	H37Rv annotation (NC_000962.3)
2195989	-	2197350	H37Rv annotation (NC_000962.3)
2330147	-	2330225	H37Rv annotation (NC_000962.3)
2365414	-	2365441	H37Rv annotation (NC_000962.3)
2366741	-	2366768	H37Rv annotation (NC_000962.3)
2372437	-	2372492	H37Rv annotation (NC_000962.3)
2372494	-	2372549	H37Rv annotation (NC_000962.3)
2430117	-	2430144	H37Rv annotation (NC_000962.3)
2431444	-	2431471	H37Rv annotation (NC_000962.3)
2458392	-	2458449	H37Rv annotation (NC_000962.3)
2493801	-	2493818	H37Rv annotation (NC_000962.3)
2522173	-	2522230	H37Rv annotation (NC_000962.3)
2523184	-	2523236	H37Rv annotation (NC_000962.3)
2531898	-	2531950	H37Rv annotation (NC_000962.3)
2531951	-	2532003	H37Rv annotation (NC_000962.3)

2532004	-	2532056	H37Rv annotation (NC_000962.3)
2532057	-	2532109	H37Rv annotation (NC_000962.3)
2532110	-	2532162	H37Rv annotation (NC_000962.3)
2532163	-	2532212	H37Rv annotation (NC_000962.3)
2550011	-	2550013	H37Rv annotation (NC_000962.3)
2550014	-	2550041	H37Rv annotation (NC_000962.3)
2551341	-	2551368	H37Rv annotation (NC_000962.3)
2551369	-	2551371	H37Rv annotation (NC_000962.3)
2635577	-	2635604	H37Rv annotation (NC_000962.3)
2636904	-	2636931	H37Rv annotation (NC_000962.3)
2687128	-	2687179	H37Rv annotation (NC_000962.3)
2687180	-	2687257	H37Rv annotation (NC_000962.3)
2716315	-	2716391	H37Rv annotation (NC_000962.3)
2720644	-	2720656	H37Rv annotation (NC_000962.3)
2721844	-	2721856	H37Rv annotation (NC_000962.3)
2762762	-	2763061	H37Rv annotation (NC_000962.3)
2763397	-	2763696	H37Rv annotation (NC_000962.3)
2784614	-	2784642	H37Rv annotation (NC_000962.3)
2785942	-	2785970	H37Rv annotation (NC_000962.3)
2800671	-	2800918	H37Rv annotation (NC_000962.3)
2806368	-	2806625	H37Rv annotation (NC_000962.3)
2972106	-	2972108	H37Rv annotation (NC_000962.3)
2972109	-	2972136	H37Rv annotation (NC_000962.3)
2973436	-	2973463	H37Rv annotation (NC_000962.3)
2973464	-	2973466	H37Rv annotation (NC_000962.3)
2983019	-	2983033	H37Rv annotation (NC_000962.3)
2996003	-	2996053	H37Rv annotation (NC_000962.3)
2996054	-	2996104	H37Rv annotation (NC_000962.3)
2996105	-	2996155	H37Rv annotation (NC_000962.3)
3007063	-	3007115	H37Rv annotation (NC_000962.3)
3007116	-	3007168	H37Rv annotation (NC_000962.3)
3007169	-	3007221	H37Rv annotation (NC_000962.3)
3013612	-	3013687	H37Rv annotation (NC_000962.3)
3073055	-	3073112	H37Rv annotation (NC_000962.3)
3119185	-	3123576	H37Rv annotation (NC_000962.3)
3119185	-	3119220	H37Rv annotation (NC_000962.3)
3119259	-	3119294	H37Rv annotation (NC_000962.3)
3119335	-	3119370	H37Rv annotation (NC_000962.3)
3119411	-	3119446	H37Rv annotation (NC_000962.3)
3119484	-	3119519	H37Rv annotation (NC_000962.3)
3119556	-	3119591	H37Rv annotation (NC_000962.3)
3119627	-	3119662	H37Rv annotation (NC_000962.3)
3119701	-	3119736	H37Rv annotation (NC_000962.3)
3119777	-	3119812	H37Rv annotation (NC_000962.3)
3119848	-	3119883	H37Rv annotation (NC_000962.3)
3119921	-	3119956	H37Rv annotation (NC_000962.3)
3119995	-	3120030	H37Rv annotation (NC_000962.3)
3120068	-	3120103	H37Rv annotation (NC_000962.3)
3120141	-	3120176	H37Rv annotation (NC_000962.3)
3120213	-	3120248	H37Rv annotation (NC_000962.3)
3120285	-	3120320	H37Rv annotation (NC_000962.3)
3120359	-	3120394	H37Rv annotation (NC_000962.3)
3120433	-	3120468	H37Rv annotation (NC_000962.3)
3120504	-	3120523	H37Rv annotation (NC_000962.3)
3121882	-	3121897	H37Rv annotation (NC_000962.3)
3121938	-	3121973	H37Rv annotation (NC_000962.3)
3122013	-	3122048	H37Rv annotation (NC_000962.3)
3122086	-	3122121	H37Rv annotation (NC_000962.3)

3122158	-	3122193	H37Rv annotation (NC_000962.3)
3122230	-	3122265	H37Rv annotation (NC_000962.3)
3122303	-	3122338	H37Rv annotation (NC_000962.3)
3122375	-	3122410	H37Rv annotation (NC_000962.3)
3122436	-	3122471	H37Rv annotation (NC_000962.3)
3122513	-	3122548	H37Rv annotation (NC_000962.3)
3122585	-	3122620	H37Rv annotation (NC_000962.3)
3122661	-	3122696	H37Rv annotation (NC_000962.3)
3122738	-	3122773	H37Rv annotation (NC_000962.3)
3122811	-	3122846	H37Rv annotation (NC_000962.3)
3122882	-	3122917	H37Rv annotation (NC_000962.3)
3122955	-	3122990	H37Rv annotation (NC_000962.3)
3123029	-	3123064	H37Rv annotation (NC_000962.3)
3123102	-	3123137	H37Rv annotation (NC_000962.3)
3123173	-	3123208	H37Rv annotation (NC_000962.3)
3123248	-	3123283	H37Rv annotation (NC_000962.3)
3123318	-	3123353	H37Rv annotation (NC_000962.3)
3123390	-	3123425	H37Rv annotation (NC_000962.3)
3123467	-	3123502	H37Rv annotation (NC_000962.3)
3123541	-	3123576	H37Rv annotation (NC_000962.3)
3155874	-	3155927	H37Rv annotation (NC_000962.3)
3155928	-	3155981	H37Rv annotation (NC_000962.3)
3155982	-	3156035	H37Rv annotation (NC_000962.3)
3156036	-	3156089	H37Rv annotation (NC_000962.3)
3160522	-	3160583	H37Rv annotation (NC_000962.3)
3171468	-	3171518	H37Rv annotation (NC_000962.3)
3171522	-	3171572	H37Rv annotation (NC_000962.3)
3171576	-	3171616	H37Rv annotation (NC_000962.3)
3181794	-	3181836	H37Rv annotation (NC_000962.3)
3192202	-	3192254	H37Rv annotation (NC_000962.3)
3192255	-	3192307	H37Rv annotation (NC_000962.3)
3192308	-	3192360	H37Rv annotation (NC_000962.3)
3318835	-	3318889	H37Rv annotation (NC_000962.3)
3319468	-	3319568	H37Rv annotation (NC_000962.3)
3319569	-	3319666	H37Rv annotation (NC_000962.3)
3333768	-	3333773	H37Rv annotation (NC_000962.3)
3335787	-	3335792	H37Rv annotation (NC_000962.3)
3381351	-	3381365	H37Rv annotation (NC_000962.3)
3382660	-	3382674	H37Rv annotation (NC_000962.3)
3481399	-	3481413	H37Rv annotation (NC_000962.3)
3482708	-	3482722	H37Rv annotation (NC_000962.3)
3551227	-	3551229	H37Rv annotation (NC_000962.3)
3551230	-	3551257	H37Rv annotation (NC_000962.3)
3552557	-	3552584	H37Rv annotation (NC_000962.3)
3552585	-	3552587	H37Rv annotation (NC_000962.3)
3552710	-	3552712	H37Rv annotation (NC_000962.3)
3552713	-	3552740	H37Rv annotation (NC_000962.3)
3554040	-	3554067	H37Rv annotation (NC_000962.3)
3554068	-	3554070	H37Rv annotation (NC_000962.3)
3591493	-	3591569	H37Rv annotation (NC_000962.3)
3626614	-	3626666	H37Rv annotation (NC_000962.3)
3658658	-	3658715	H37Rv annotation (NC_000962.3)
3704895	-	3705004	H37Rv annotation (NC_000962.3)
3710382	-	3710409	H37Rv annotation (NC_000962.3)
3711709	-	3711736	H37Rv annotation (NC_000962.3)
3743198	-	3743404	H37Rv annotation (NC_000962.3)
3743402	-	3743510	H37Rv annotation (NC_000962.3)
3743508	-	3743605	H37Rv annotation (NC_000962.3)

3769514	-	3769720	H37Rv annotation (NC_000962.3)
3769754	-	3769862	H37Rv annotation (NC_000962.3)
3770994	-	3771091	H37Rv annotation (NC_000962.3)
3795058	-	3795085	H37Rv annotation (NC_000962.3)
3796385	-	3796412	H37Rv annotation (NC_000962.3)
3799987	-	3800011	H37Rv annotation (NC_000962.3)
3801530	-	3801554	H37Rv annotation (NC_000962.3)
3883550	-	3884921	H37Rv annotation (NC_000962.3)
3890779	-	3890806	H37Rv annotation (NC_000962.3)
3892106	-	3892133	H37Rv annotation (NC_000962.3)
3945098	-	3945597	H37Rv annotation (NC_000962.3)
3950830	-	3951329	H37Rv annotation (NC_000962.3)
3991568	-	3991625	H37Rv annotation (NC_000962.3)
4052949	-	4052966	H37Rv annotation (NC_000962.3)
4052971	-	4052994	H37Rv annotation (NC_000962.3)
4052995	-	4053105	H37Rv annotation (NC_000962.3)
4053004	-	4053021	H37Rv annotation (NC_000962.3)
4053106	-	4053216	H37Rv annotation (NC_000962.3)
4053217	-	4053327	H37Rv annotation (NC_000962.3)
4053328	-	4053438	H37Rv annotation (NC_000962.3)
4053439	-	4053549	H37Rv annotation (NC_000962.3)
4075615	-	4075630	H37Rv annotation (NC_000962.3)
4077735	-	4077750	H37Rv annotation (NC_000962.3)
4078506	-	4078518	H37Rv annotation (NC_000962.3)
4079786	-	4079798	H37Rv annotation (NC_000962.3)
4134601	-	4134725	H37Rv annotation (NC_000962.3)
4348721	-	4348773	H37Rv annotation (NC_000962.3)
4348774	-	4348826	H37Rv annotation (NC_000962.3)
4353280	-	4353330	H37Rv annotation (NC_000962.3)
4353331	-	4353381	H37Rv annotation (NC_000962.3)
4353382	-	4353432	H37Rv annotation (NC_000962.3)
80185	-	80373	NUCmer repeat finding (this study)
80927	-	82664	NUCmer repeat finding (this study)
103706	-	105076	NUCmer repeat finding (this study)
103780	-	105130	NUCmer repeat finding (this study)
149572	-	149762	NUCmer repeat finding (this study)
154115	-	154186	NUCmer repeat finding (this study)
154168	-	154239	NUCmer repeat finding (this study)
177055	-	177175	NUCmer repeat finding (this study)
177169	-	177451	NUCmer repeat finding (this study)
197824	-	198205	NUCmer repeat finding (this study)
309030	-	309532	NUCmer repeat finding (this study)
309899	-	310101	NUCmer repeat finding (this study)
332699	-	333610	NUCmer repeat finding (this study)
333511	-	333734	NUCmer repeat finding (this study)
335829	-	336275	NUCmer repeat finding (this study)
335417	-	336379	NUCmer repeat finding (this study)
336566	-	336788	NUCmer repeat finding (this study)
338214	-	338450	NUCmer repeat finding (this study)
338298	-	338537	NUCmer repeat finding (this study)
338201	-	339142	NUCmer repeat finding (this study)
339373	-	339875	NUCmer repeat finding (this study)
366867	-	367146	NUCmer repeat finding (this study)
368076	-	368602	NUCmer repeat finding (this study)
369390	-	369805	NUCmer repeat finding (this study)
370548	-	370963	NUCmer repeat finding (this study)
371821	-	372375	NUCmer repeat finding (this study)
372246	-	372525	NUCmer repeat finding (this study)

374567	-	374856	NUCmer repeat finding (this study)
374504	-	375058	NUCmer repeat finding (this study)
374593	-	375119	NUCmer repeat finding (this study)
400152	-	401701	NUCmer repeat finding (this study)
428385	-	428757	NUCmer repeat finding (this study)
428368	-	428760	NUCmer repeat finding (this study)
429620	-	429909	NUCmer repeat finding (this study)
424012	-	429920	NUCmer repeat finding (this study)
429766	-	430167	NUCmer repeat finding (this study)
429648	-	430190	NUCmer repeat finding (this study)
429662	-	430194	NUCmer repeat finding (this study)
431159	-	431541	NUCmer repeat finding (this study)
431025	-	431567	NUCmer repeat finding (this study)
432541	-	432929	NUCmer repeat finding (this study)
432401	-	432933	NUCmer repeat finding (this study)
460808	-	461483	NUCmer repeat finding (this study)
473744	-	474099	NUCmer repeat finding (this study)
475810	-	476177	NUCmer repeat finding (this study)
562307	-	562394	NUCmer repeat finding (this study)
569821	-	569922	NUCmer repeat finding (this study)
569877	-	569978	NUCmer repeat finding (this study)
577286	-	577438	NUCmer repeat finding (this study)
577344	-	577496	NUCmer repeat finding (this study)
580577	-	580737	NUCmer repeat finding (this study)
580654	-	580814	NUCmer repeat finding (this study)
606511	-	608060	NUCmer repeat finding (this study)
675486	-	675612	NUCmer repeat finding (this study)
675492	-	675653	NUCmer repeat finding (this study)
686110	-	686491	NUCmer repeat finding (this study)
798824	-	799109	NUCmer repeat finding (this study)
832045	-	832356	NUCmer repeat finding (this study)
838771	-	838896	NUCmer repeat finding (this study)
838486	-	838926	NUCmer repeat finding (this study)
840739	-	841665	NUCmer repeat finding (this study)
842020	-	842331	NUCmer repeat finding (this study)
889020	-	890375	NUCmer repeat finding (this study)
889021	-	891461	NUCmer repeat finding (this study)
908181	-	909346	NUCmer repeat finding (this study)
917611	-	917677	NUCmer repeat finding (this study)
917669	-	917735	NUCmer repeat finding (this study)
921744	-	921892	NUCmer repeat finding (this study)
921580	-	921893	NUCmer repeat finding (this study)
925280	-	925404	NUCmer repeat finding (this study)
960167	-	960270	NUCmer repeat finding (this study)
960213	-	960323	NUCmer repeat finding (this study)
960228	-	960324	NUCmer repeat finding (this study)
960175	-	960325	NUCmer repeat finding (this study)
960220	-	960327	NUCmer repeat finding (this study)
964605	-	964965	NUCmer repeat finding (this study)
964770	-	965086	NUCmer repeat finding (this study)
1093336	-	1093666	NUCmer repeat finding (this study)
1093422	-	1093866	NUCmer repeat finding (this study)
1093841	-	1094592	NUCmer repeat finding (this study)
1095120	-	1095317	NUCmer repeat finding (this study)
1095175	-	1095393	NUCmer repeat finding (this study)
1095088	-	1095413	NUCmer repeat finding (this study)
1095372	-	1095566	NUCmer repeat finding (this study)
1095301	-	1095739	NUCmer repeat finding (this study)

1095936	-	1096687	NUCmer repeat finding (this study)
1095821	-	1096692	NUCmer repeat finding (this study)
1160544	-	1161151	NUCmer repeat finding (this study)
1160539	-	1161165	NUCmer repeat finding (this study)
1160546	-	1161167	NUCmer repeat finding (this study)
1164572	-	1165549	NUCmer repeat finding (this study)
1169297	-	1170206	NUCmer repeat finding (this study)
1169298	-	1170733	NUCmer repeat finding (this study)
1189340	-	1189539	NUCmer repeat finding (this study)
1190000	-	1190120	NUCmer repeat finding (this study)
1189730	-	1190175	NUCmer repeat finding (this study)
1191259	-	1191467	NUCmer repeat finding (this study)
1191724	-	1191882	NUCmer repeat finding (this study)
1191454	-	1191899	NUCmer repeat finding (this study)
1211532	-	1212158	NUCmer repeat finding (this study)
1216792	-	1217061	NUCmer repeat finding (this study)
1216441	-	1217067	NUCmer repeat finding (this study)
1275925	-	1276053	NUCmer repeat finding (this study)
1276293	-	1277797	NUCmer repeat finding (this study)
1277843	-	1278820	NUCmer repeat finding (this study)
1287355	-	1288094	NUCmer repeat finding (this study)
1305495	-	1305599	NUCmer repeat finding (this study)
1305557	-	1305661	NUCmer repeat finding (this study)
1339350	-	1340433	NUCmer repeat finding (this study)
1340642	-	1341288	NUCmer repeat finding (this study)
1340495	-	1341292	NUCmer repeat finding (this study)
1341296	-	1342730	NUCmer repeat finding (this study)
1357423	-	1357613	NUCmer repeat finding (this study)
1441366	-	1441638	NUCmer repeat finding (this study)
1441519	-	1441791	NUCmer repeat finding (this study)
1451779	-	1451994	NUCmer repeat finding (this study)
1451836	-	1451995	NUCmer repeat finding (this study)
1451778	-	1451998	NUCmer repeat finding (this study)
1468166	-	1469616	NUCmer repeat finding (this study)
1479356	-	1480072	NUCmer repeat finding (this study)
1481030	-	1481746	NUCmer repeat finding (this study)
1532540	-	1533653	NUCmer repeat finding (this study)
1541949	-	1543307	NUCmer repeat finding (this study)
1541952	-	1543318	NUCmer repeat finding (this study)
1633311	-	1633538	NUCmer repeat finding (this study)
1633531	-	1634790	NUCmer repeat finding (this study)
1636706	-	1636933	NUCmer repeat finding (this study)
1637133	-	1638392	NUCmer repeat finding (this study)
1644263	-	1644365	NUCmer repeat finding (this study)
1702677	-	1702843	NUCmer repeat finding (this study)
1702707	-	1702849	NUCmer repeat finding (this study)
1773962	-	1774031	NUCmer repeat finding (this study)
1774037	-	1774106	NUCmer repeat finding (this study)
1779809	-	1780054	NUCmer repeat finding (this study)
1779984	-	1780311	NUCmer repeat finding (this study)
1780241	-	1780580	NUCmer repeat finding (this study)
1780641	-	1782817	NUCmer repeat finding (this study)
1785877	-	1786318	NUCmer repeat finding (this study)
1788514	-	1789865	NUCmer repeat finding (this study)
1814895	-	1815098	NUCmer repeat finding (this study)
1814853	-	1815128	NUCmer repeat finding (this study)
1876112	-	1876668	NUCmer repeat finding (this study)
1875622	-	1876899	NUCmer repeat finding (this study)

1879291	-	1879378	NUCmer repeat finding (this study)
1882019	-	1883293	NUCmer repeat finding (this study)
1895353	-	1895534	NUCmer repeat finding (this study)
1895410	-	1895591	NUCmer repeat finding (this study)
1946849	-	1947023	NUCmer repeat finding (this study)
1963420	-	1964160	NUCmer repeat finding (this study)
1982887	-	1983273	NUCmer repeat finding (this study)
1983043	-	1983350	NUCmer repeat finding (this study)
1982013	-	1983351	NUCmer repeat finding (this study)
1987699	-	1989057	NUCmer repeat finding (this study)
1987702	-	1989058	NUCmer repeat finding (this study)
1987703	-	1989059	NUCmer repeat finding (this study)
1996101	-	1997456	NUCmer repeat finding (this study)
1996100	-	1997458	NUCmer repeat finding (this study)
1996099	-	1997463	NUCmer repeat finding (this study)
1997453	-	1998612	NUCmer repeat finding (this study)
2000927	-	2001050	NUCmer repeat finding (this study)
2000930	-	2001056	NUCmer repeat finding (this study)
2025302	-	2026163	NUCmer repeat finding (this study)
2028426	-	2029242	NUCmer repeat finding (this study)
2030347	-	2030978	NUCmer repeat finding (this study)
2030332	-	2030983	NUCmer repeat finding (this study)
2059430	-	2059523	NUCmer repeat finding (this study)
2059507	-	2059600	NUCmer repeat finding (this study)
2062007	-	2062263	NUCmer repeat finding (this study)
2062400	-	2062639	NUCmer repeat finding (this study)
2074436	-	2074577	NUCmer repeat finding (this study)
2074492	-	2074633	NUCmer repeat finding (this study)
2089244	-	2089483	NUCmer repeat finding (this study)
2134912	-	2135764	NUCmer repeat finding (this study)
2163328	-	2163461	NUCmer repeat finding (this study)
2163397	-	2163530	NUCmer repeat finding (this study)
2163731	-	2164023	NUCmer repeat finding (this study)
2163800	-	2164092	NUCmer repeat finding (this study)
2165327	-	2165508	NUCmer repeat finding (this study)
2165402	-	2165583	NUCmer repeat finding (this study)
2195857	-	2197360	NUCmer repeat finding (this study)
2198687	-	2198815	NUCmer repeat finding (this study)
2262010	-	2263169	NUCmer repeat finding (this study)
2294817	-	2296422	NUCmer repeat finding (this study)
2299556	-	2300118	NUCmer repeat finding (this study)
2299197	-	2300822	NUCmer repeat finding (this study)
2300913	-	2302512	NUCmer repeat finding (this study)
2305625	-	2306187	NUCmer repeat finding (this study)
2305266	-	2306889	NUCmer repeat finding (this study)
2337937	-	2338094	NUCmer repeat finding (this study)
2340530	-	2340687	NUCmer repeat finding (this study)
2347380	-	2347554	NUCmer repeat finding (this study)
2347391	-	2347611	NUCmer repeat finding (this study)
2347452	-	2347613	NUCmer repeat finding (this study)
2358503	-	2359351	NUCmer repeat finding (this study)
2359727	-	2359994	NUCmer repeat finding (this study)
2365404	-	2366768	NUCmer repeat finding (this study)
2365414	-	2366769	NUCmer repeat finding (this study)
2365411	-	2366771	NUCmer repeat finding (this study)
2430115	-	2431471	NUCmer repeat finding (this study)
2430116	-	2431472	NUCmer repeat finding (this study)
2430117	-	2431473	NUCmer repeat finding (this study)

2430114	-	2431474	NUCmer repeat finding (this study)
2439140	-	2439948	NUCmer repeat finding (this study)
2461320	-	2461447	NUCmer repeat finding (this study)
2461378	-	2461504	NUCmer repeat finding (this study)
2531900	-	2532002	NUCmer repeat finding (this study)
2531893	-	2532156	NUCmer repeat finding (this study)
2531946	-	2532209	NUCmer repeat finding (this study)
2550012	-	2551368	NUCmer repeat finding (this study)
2550006	-	2551369	NUCmer repeat finding (this study)
2550010	-	2551370	NUCmer repeat finding (this study)
2625890	-	2626536	NUCmer repeat finding (this study)
2625886	-	2626683	NUCmer repeat finding (this study)
2635145	-	2635576	NUCmer repeat finding (this study)
2635575	-	2636931	NUCmer repeat finding (this study)
2635577	-	2636932	NUCmer repeat finding (this study)
2635576	-	2636937	NUCmer repeat finding (this study)
2636930	-	2637028	NUCmer repeat finding (this study)
2637300	-	2637501	NUCmer repeat finding (this study)
2637258	-	2637533	NUCmer repeat finding (this study)
2637164	-	2637535	NUCmer repeat finding (this study)
2638293	-	2638724	NUCmer repeat finding (this study)
2639035	-	2639531	NUCmer repeat finding (this study)
2639515	-	2639613	NUCmer repeat finding (this study)
2720635	-	2721442	NUCmer repeat finding (this study)
2784601	-	2785969	NUCmer repeat finding (this study)
2784615	-	2785970	NUCmer repeat finding (this study)
2784614	-	2785977	NUCmer repeat finding (this study)
2829020	-	2829928	NUCmer repeat finding (this study)
2828494	-	2829930	NUCmer repeat finding (this study)
2866469	-	2867121	NUCmer repeat finding (this study)
2867125	-	2867777	NUCmer repeat finding (this study)
2882593	-	2884330	NUCmer repeat finding (this study)
2944470	-	2945221	NUCmer repeat finding (this study)
2944354	-	2945226	NUCmer repeat finding (this study)
2971757	-	2971987	NUCmer repeat finding (this study)
2972105	-	2973463	NUCmer repeat finding (this study)
2973793	-	2975987	NUCmer repeat finding (this study)
2978623	-	2979060	NUCmer repeat finding (this study)
2982945	-	2983854	NUCmer repeat finding (this study)
2996010	-	2996104	NUCmer repeat finding (this study)
2996004	-	2996117	NUCmer repeat finding (this study)
2996011	-	2996155	NUCmer repeat finding (this study)
2996055	-	2996168	NUCmer repeat finding (this study)
3000741	-	3001893	NUCmer repeat finding (this study)
3002110	-	3003262	NUCmer repeat finding (this study)
3007038	-	3007167	NUCmer repeat finding (this study)
3007091	-	3007220	NUCmer repeat finding (this study)
3082466	-	3082654	NUCmer repeat finding (this study)
3100174	-	3100323	NUCmer repeat finding (this study)
3100175	-	3102010	NUCmer repeat finding (this study)
3100486	-	3102227	NUCmer repeat finding (this study)
3112884	-	3113179	NUCmer repeat finding (this study)
3113642	-	3113937	NUCmer repeat finding (this study)
3116388	-	3117192	NUCmer repeat finding (this study)
3119183	-	3120015	NUCmer repeat finding (this study)
3119333	-	3120103	NUCmer repeat finding (this study)
3119334	-	3120248	NUCmer repeat finding (this study)
3119938	-	3120464	NUCmer repeat finding (this study)

3119185	-	3120524	NUCmer repeat finding (this study)
3120523	-	3121879	NUCmer repeat finding (this study)
3120518	-	3121880	NUCmer repeat finding (this study)
3120521	-	3121881	NUCmer repeat finding (this study)
3120522	-	3121882	NUCmer repeat finding (this study)
3121934	-	3122406	NUCmer repeat finding (this study)
3122431	-	3122831	NUCmer repeat finding (this study)
3122012	-	3123208	NUCmer repeat finding (this study)
3122013	-	3123338	NUCmer repeat finding (this study)
3121937	-	3123354	NUCmer repeat finding (this study)
3121879	-	3123502	NUCmer repeat finding (this study)
3122230	-	3123561	NUCmer repeat finding (this study)
3123097	-	3123572	NUCmer repeat finding (this study)
3122011	-	3123576	NUCmer repeat finding (this study)
3122229	-	3123577	NUCmer repeat finding (this study)
3122436	-	3123581	NUCmer repeat finding (this study)
3132892	-	3133455	NUCmer repeat finding (this study)
3135788	-	3136351	NUCmer repeat finding (this study)
3155883	-	3156088	NUCmer repeat finding (this study)
3155937	-	3156089	NUCmer repeat finding (this study)
3171468	-	3171572	NUCmer repeat finding (this study)
3171463	-	3171607	NUCmer repeat finding (this study)
3171466	-	3171622	NUCmer repeat finding (this study)
3171522	-	3171626	NUCmer repeat finding (this study)
3171525	-	3171627	NUCmer repeat finding (this study)
3189482	-	3189567	NUCmer repeat finding (this study)
3189539	-	3189624	NUCmer repeat finding (this study)
3192195	-	3192300	NUCmer repeat finding (this study)
3192257	-	3192352	NUCmer repeat finding (this study)
3192248	-	3192353	NUCmer repeat finding (this study)
3192204	-	3192354	NUCmer repeat finding (this study)
3194138	-	3194451	NUCmer repeat finding (this study)
3194139	-	3195506	NUCmer repeat finding (this study)
3232651	-	3232810	NUCmer repeat finding (this study)
3232653	-	3232865	NUCmer repeat finding (this study)
3232707	-	3232866	NUCmer repeat finding (this study)
3239479	-	3239582	NUCmer repeat finding (this study)
3239471	-	3239743	NUCmer repeat finding (this study)
3239526	-	3239829	NUCmer repeat finding (this study)
3247062	-	3248167	NUCmer repeat finding (this study)
3252477	-	3253581	NUCmer repeat finding (this study)
3317534	-	3317739	NUCmer repeat finding (this study)
3319469	-	3319558	NUCmer repeat finding (this study)
3319570	-	3319657	NUCmer repeat finding (this study)
3334063	-	3334386	NUCmer repeat finding (this study)
3333758	-	3335596	NUCmer repeat finding (this study)
3336504	-	3336744	NUCmer repeat finding (this study)
3336563	-	3336802	NUCmer repeat finding (this study)
3351091	-	3351158	NUCmer repeat finding (this study)
3351147	-	3351214	NUCmer repeat finding (this study)
3376927	-	3377391	NUCmer repeat finding (this study)
3377649	-	3378269	NUCmer repeat finding (this study)
3378333	-	3378450	NUCmer repeat finding (this study)
3379364	-	3379828	NUCmer repeat finding (this study)
3380089	-	3380708	NUCmer repeat finding (this study)
3380911	-	3381026	NUCmer repeat finding (this study)
3381312	-	3382747	NUCmer repeat finding (this study)
3381313	-	3382749	NUCmer repeat finding (this study)

3481325	-	3482760	NUCmer repeat finding (this study)
3483946	-	3485111	NUCmer repeat finding (this study)
3528675	-	3529162	NUCmer repeat finding (this study)
3551225	-	3552584	NUCmer repeat finding (this study)
3551230	-	3552585	NUCmer repeat finding (this study)
3551227	-	3552587	NUCmer repeat finding (this study)
3552705	-	3554067	NUCmer repeat finding (this study)
3552713	-	3554068	NUCmer repeat finding (this study)
3552709	-	3554070	NUCmer repeat finding (this study)
3594263	-	3594402	NUCmer repeat finding (this study)
3594319	-	3594458	NUCmer repeat finding (this study)
3663699	-	3663825	NUCmer repeat finding (this study)
3663860	-	3663987	NUCmer repeat finding (this study)
3653792	-	3663995	NUCmer repeat finding (this study)
3710381	-	3711737	NUCmer repeat finding (this study)
3710380	-	3711738	NUCmer repeat finding (this study)
3710382	-	3712822	NUCmer repeat finding (this study)
3729364	-	3729681	NUCmer repeat finding (this study)
3730353	-	3731031	NUCmer repeat finding (this study)
3730351	-	3731064	NUCmer repeat finding (this study)
3732081	-	3732759	NUCmer repeat finding (this study)
3732079	-	3732792	NUCmer repeat finding (this study)
3735636	-	3736314	NUCmer repeat finding (this study)
3743198	-	3743501	NUCmer repeat finding (this study)
3743508	-	3743602	NUCmer repeat finding (this study)
3743711	-	3744009	NUCmer repeat finding (this study)
3743710	-	3744269	NUCmer repeat finding (this study)
3744460	-	3745336	NUCmer repeat finding (this study)
3744704	-	3745407	NUCmer repeat finding (this study)
3745500	-	3746006	NUCmer repeat finding (this study)
3746513	-	3747213	NUCmer repeat finding (this study)
3746540	-	3747233	NUCmer repeat finding (this study)
3746507	-	3747571	NUCmer repeat finding (this study)
3746616	-	3747586	NUCmer repeat finding (this study)
3746511	-	3747596	NUCmer repeat finding (this study)
3746303	-	3747763	NUCmer repeat finding (this study)
3748283	-	3748956	NUCmer repeat finding (this study)
3748322	-	3749015	NUCmer repeat finding (this study)
3748141	-	3749092	NUCmer repeat finding (this study)
3750167	-	3750773	NUCmer repeat finding (this study)
3750140	-	3750802	NUCmer repeat finding (this study)
3750137	-	3750862	NUCmer repeat finding (this study)
3750241	-	3751192	NUCmer repeat finding (this study)
3750134	-	3751201	NUCmer repeat finding (this study)
3751635	-	3753178	NUCmer repeat finding (this study)
3753331	-	3754361	NUCmer repeat finding (this study)
3755952	-	3756266	NUCmer repeat finding (this study)
3755951	-	3756510	NUCmer repeat finding (this study)
3756701	-	3757577	NUCmer repeat finding (this study)
3756933	-	3757609	NUCmer repeat finding (this study)
3757046	-	3757972	NUCmer repeat finding (this study)
3756942	-	3758031	NUCmer repeat finding (this study)
3757711	-	3758217	NUCmer repeat finding (this study)
3758734	-	3759401	NUCmer repeat finding (this study)
3758703	-	3759792	NUCmer repeat finding (this study)
3758496	-	3759959	NUCmer repeat finding (this study)
3760476	-	3761152	NUCmer repeat finding (this study)
3760516	-	3761183	NUCmer repeat finding (this study)

3760334	-	3761288	NUCmer repeat finding (this study)
3762386	-	3763337	NUCmer repeat finding (this study)
3762388	-	3763361	NUCmer repeat finding (this study)
3764037	-	3764678	NUCmer repeat finding (this study)
3764044	-	3765073	NUCmer repeat finding (this study)
3764043	-	3765086	NUCmer repeat finding (this study)
3765550	-	3767096	NUCmer repeat finding (this study)
3769514	-	3769853	NUCmer repeat finding (this study)
3770994	-	3771088	NUCmer repeat finding (this study)
3795044	-	3796412	NUCmer repeat finding (this study)
3795055	-	3796416	NUCmer repeat finding (this study)
3801958	-	3802230	NUCmer repeat finding (this study)
3802130	-	3802389	NUCmer repeat finding (this study)
3820393	-	3820473	NUCmer repeat finding (this study)
3820391	-	3820532	NUCmer repeat finding (this study)
3820448	-	3820589	NUCmer repeat finding (this study)
3841533	-	3842766	NUCmer repeat finding (this study)
3842149	-	3842904	NUCmer repeat finding (this study)
3842947	-	3843702	NUCmer repeat finding (this study)
3846461	-	3847692	NUCmer repeat finding (this study)
3847717	-	3847892	NUCmer repeat finding (this study)
3883550	-	3884921	NUCmer repeat finding (this study)
3890772	-	3892133	NUCmer repeat finding (this study)
3890777	-	3892135	NUCmer repeat finding (this study)
3890778	-	3892140	NUCmer repeat finding (this study)
3894406	-	3895519	NUCmer repeat finding (this study)
3930989	-	3932758	NUCmer repeat finding (this study)
3932294	-	3932794	NUCmer repeat finding (this study)
3932789	-	3933043	NUCmer repeat finding (this study)
3932676	-	3933045	NUCmer repeat finding (this study)
3933381	-	3933560	NUCmer repeat finding (this study)
3933402	-	3933562	NUCmer repeat finding (this study)
3933392	-	3934165	NUCmer repeat finding (this study)
3933995	-	3934768	NUCmer repeat finding (this study)
3934655	-	3934894	NUCmer repeat finding (this study)
3935229	-	3935411	NUCmer repeat finding (this study)
3935300	-	3935557	NUCmer repeat finding (this study)
3934871	-	3935806	NUCmer repeat finding (this study)
3935844	-	3936026	NUCmer repeat finding (this study)
3935915	-	3936172	NUCmer repeat finding (this study)
3935486	-	3936415	NUCmer repeat finding (this study)
3941225	-	3941626	NUCmer repeat finding (this study)
3941561	-	3941965	NUCmer repeat finding (this study)
3945098	-	3945597	NUCmer repeat finding (this study)
3946946	-	3947301	NUCmer repeat finding (this study)
3946945	-	3947491	NUCmer repeat finding (this study)
3945778	-	3947535	NUCmer repeat finding (this study)
3946934	-	3947652	NUCmer repeat finding (this study)
3947287	-	3947842	NUCmer repeat finding (this study)
3947276	-	3948003	NUCmer repeat finding (this study)
3947639	-	3948005	NUCmer repeat finding (this study)
3947990	-	3948082	NUCmer repeat finding (this study)
3947989	-	3948243	NUCmer repeat finding (this study)
3948218	-	3948399	NUCmer repeat finding (this study)
3947881	-	3948433	NUCmer repeat finding (this study)
3948013	-	3948507	NUCmer repeat finding (this study)
3948366	-	3948655	NUCmer repeat finding (this study)
3948610	-	3948727	NUCmer repeat finding (this study)

3948941	-	3949033	NUCmer repeat finding (this study)
3948312	-	3949401	NUCmer repeat finding (this study)
3949569	-	3949864	NUCmer repeat finding (this study)
3949750	-	3949872	NUCmer repeat finding (this study)
3948912	-	3950001	NUCmer repeat finding (this study)
3950830	-	3951329	NUCmer repeat finding (this study)
4039486	-	4040155	NUCmer repeat finding (this study)
4052971	-	4053438	NUCmer repeat finding (this study)
4053082	-	4053549	NUCmer repeat finding (this study)
4053634	-	4053900	NUCmer repeat finding (this study)
4053632	-	4053994	NUCmer repeat finding (this study)
4059984	-	4060591	NUCmer repeat finding (this study)
4078002	-	4078200	NUCmer repeat finding (this study)
4077999	-	4078201	NUCmer repeat finding (this study)
4087480	-	4087562	NUCmer repeat finding (this study)
4087537	-	4087619	NUCmer repeat finding (this study)
4120917	-	4120996	NUCmer repeat finding (this study)
4120974	-	4121053	NUCmer repeat finding (this study)
4155460	-	4155525	NUCmer repeat finding (this study)
4155517	-	4155582	NUCmer repeat finding (this study)
4156799	-	4156908	NUCmer repeat finding (this study)
4156858	-	4156967	NUCmer repeat finding (this study)
4215915	-	4216724	NUCmer repeat finding (this study)
4221089	-	4221937	NUCmer repeat finding (this study)
4222268	-	4222535	NUCmer repeat finding (this study)
4252882	-	4254332	NUCmer repeat finding (this study)
4265694	-	4266546	NUCmer repeat finding (this study)
4288136	-	4288256	NUCmer repeat finding (this study)
4301542	-	4301691	NUCmer repeat finding (this study)
4301685	-	4302008	NUCmer repeat finding (this study)
4301691	-	4303432	NUCmer repeat finding (this study)
4317966	-	4318157	NUCmer repeat finding (this study)
4318341	-	4319371	NUCmer repeat finding (this study)
4348725	-	4348832	NUCmer repeat finding (this study)
4353280	-	4353380	NUCmer repeat finding (this study)
4353331	-	4353431	NUCmer repeat finding (this study)
