# Supplementary material for: First insights on the genetic diversity of MDR Mycobacterium tuberculosis in Lebanon
Source: BMC Infect Dis. 2018 Dec 29;18:710. doi: 10.1186/s12879-018-3626-3 (PMC6311033; doi:10.1186/s12879-018-3626-3)
Supplement: Supplementary file 5 — Antimicrobial resistance profiles and detected mutations in the corresponding AMR genes of the TB isolates. R: Resistant; S: sensitive; +: MDR; −: Not MDR; Sensitive and resistant are indicated in green and red, respectively. (PDF 37 kb) [file 12879_2018_3626_MOESM5_ESM.pdf]

		Isoniazid		Rifampicin		Ethambutol		Quinolones			Streptomycin				Pyrazinamide							Amikacin	Capreomycin	Kanamycin	MDR
**#**	**Sample**		***katG*; S315T**		***rpoB*; S450L**		***embB*; M306V**		***gyrA*; A90X**	***gyrA*; D94X**		***rrs*; A514X**	***rpsL*; K43R**	***rpsL*;K88R**		***pncA*; H57D**	***pncA*; S65S**	***pncA*; V44A**	***pncA*; S65F**	***pncA*; S65T**	***pncA*; A46P**				
**1**	TB-4	S		S		S		S			S				S							S	S	S	-
**2**	TB-5	S		S		S		S			S				R	SNP2289073GC						S	S	S	-
**3**	TB-7	S		S		S		S			S				S							S	S	S	-
**4**	TB-8	S		S		S		S			S				R	SNP2289073GC						S	S	S	-
**5**	TB-9	S		S		S		S			S				S							S	S	S	-
**6**	TB-10	S		S		S		S			S				S							S	S	S	-
**7**	TB-12	R	SNP2155168CG	R	SNP761155CT	R	SNP4247429AC	S			R	SNP1472359AC			R		SNP2289047GA	SNP2289111AG				S	S	S	+
**8**	TB-13	S		S		S		S			S				S							S	S	S	-
**9**	TB-14	S		S		S		R	SNP7570CT		S				S							S	S	S	-
**10**	TB-15	S		S		S		S			S				S							S	S	S	-
**11**	TB-16	R	SNP2155168CG	R	SNP761155CT	R	SNP4247429AG	S			R			SNP781822AG	R				SNP2289048GA	SNP2289049AT		S	S	S	+
**12**	TB-17	R	SNP2155168CG	R	SNP761155CT	S		R		SNP7582AC	R		SNP781687AG		R						SNP2289106CG	S	S	S	+
**13**	TB-20	S		S		S		S			S				S							S	S	S	-
