# Supplementary material for: First insights on the genetic diversity of MDR Mycobacterium tuberculosis in Lebanon
Source: BMC Infect Dis. 2018 Dec 29;18:710. doi: 10.1186/s12879-018-3626-3 (PMC6311033; doi:10.1186/s12879-018-3626-3)
Supplement: Supplementary file 6 — Results of the MIRU-Profiler showing the number of tandem repeats for the 24 classical MIRU loci. The results of MIRUprofiler are based on 24 classical VNTR loci. (PDF 13 kb) [file 12879_2018_3626_MOESM6_ESM.pdf]

	TB4	TB5	TB7	TB8	TB9	TB10	TB12	TB13	TB14	TB15	TB16	TB17	TB20
MIRU02	N/A	2	2	2	2	2	2	2	2	2	2	2	2
Mtub04	1	2	1	2	1	2	2	1	1	2	2	2	2
ETRC	5	7	6	3	3	3	2	4	3	4	2	3	3
MIRU04	2	2	2	2	2	2	2	3	2	2	2	2	2
MIRU40	2	2	2	2	3	N/A	N/A	2	2	1	2	2	2
MIRU10	3	2	5	2	4	7	4	7	2	3	7	3	7
MIRU16	2	3	3	3	1	3	3	3	1	3	3	3	3
Mtub21	3	3	2	3	2	3	3	8	2	3	3	7	7
MIRU20	2	1	2	2	2	2	2	2	2	1	2	2	2
QUB11b	2	2	6	2	2	6	2	3	3	2	N/A	6	2
ETRA	3	6	2	N/A	3	3	3	4	4	3	3	3	3
Mtub29	6	3	6	3	5	2	5	3	4	7	7	7	7
Mtub30	2	7	2	3	N/A	6	2	2	2	2	2	3	7
ETRB	2	3	2	3	2	2	2	7	2	2	2	2	2
MIRU23	3	4	4	4	5	3	5	5	5	5	4	5	6
MIRU24	1	4	1	2	1	1	1	2	1	1	1	1	1
MIRU26	3	3	5	3	1	5	3	2	3	3	1	4	3
MIRU27	5	N/A	1	N/A	3	6	6	N/A	5	5	5	6	5
Mtub34	2	3	3	3	3	3	3	3	3	3	3	3	3
MIRU31	3	3	3	3	2	3	3	4	3	3	3	3	3
Mtub39	7	2	N/A	2	3	3	3	2	N/A	N/A	2	7	3
QUB26	4	2	4	2	4	5	4	5	4	4	4	5	6
QUB4156	2	1	2	1	6	4	2	1	2	2	2	2	6
MIRU39	2	1	2	2	2	2	7	N/A	1	2	3	3	2
